# Treatment of Metastatic Uveal Melanoma: Systematic Review

**DOI:** 10.3390/cancers12092557

**Published:** 2020-09-08

**Authors:** Cristina Rodriguez-Vidal, Daniel Fernandez-Diaz, Beatriz Fernandez-Marta, Nerea Lago-Baameiro, María Pardo, Paula Silva, Laura Paniagua, María José Blanco-Teijeiro, Antonio Piñeiro, Manuel Bande

**Affiliations:** 1 Department of Ophthalmology, University Hospital of Cruces, Cruces Plaza S/N, 48903 Barakaldo-Vizcaya, Spain; cristina.rodriguez.vidal@rai.usc.es; 2 Department of Ophthalmology, University Hospital of Santiago de Compostela, Ramon Baltar S/N, 15706 Santiago de Compostela, Spain; daniel.fernandez.diaz@rai.usc.es (D.F.-D.); beatriz.fernandez.marta@rai.usc.es (B.F.-M.); mariajose.blanco@usc.es (M.J.B.-T.); antonio.pineiro@usc.es (A.P.); 3 Tumores Intraoculares en el Adulto, Instituto de Investigación Sanitaria de Santiago (IDIS), 15706 Santiago de Compostela, Spain; maria.pardo.perez@sergas.es (M.P.); paula.silva.rodriguez@rai.usc.es (P.S.); 4 Grupo Obesidómica, Instituto de Investigación Sanitaria de Santiago (IDIS), 15706 Santiago de Compostela, Spain; nerea.lago@rai.usc.es; 5 Fundación Pública Galega de Medicina Xenómica, Clinical University Hospital, SERGAS, 15705 Santiago de Compostela, Spain; 6 Department of Ophthalmology, University Hospital of Coruña, Praza Parrote s/n, 15006 A Coruña, Spain; laura.paniagua@rai.usc.es

**Keywords:** uveal melanoma, metastatic, review

## Abstract

**Simple Summary:**

Contrary to other cancers, treatment of human uveal malignant melanoma metastases has not evolved sufficiently remaining as a great challenge in the field of ocular oncology. Although uveal melanoma is effectively controlled at the local level, the diagnosis of systemic disease in these patients makes its prognosis fatal, with survival rates of around 4–8 months. In this manuscript, we performed a systematic review studying comprehensively all the different treatment types for metastatic uveal melanoma disease in the last 40 years. We truly believe that our work shows a global vision of the situation, placing the reader in a concise and orderly manner in perspective of the current state of the subject.

**Abstract:**

Introduction: More than 50% of patients with uveal melanoma end up developing metastases. Currently, there is no standard first-line treatment that facilitates proper management of the metastatic disease. Methods: A systematic review of the last 40 years in PubMed with an exhaustive and strict selection of studies was conducted, in which the unit of measurement was overall survival (OS) expressed in Kaplan–Meier curves or numerically. Results: After the selection process, 110 articles were included. Regional therapies, such as intra-arterial liver chemotherapy (OS: 2, 9–22 months), isolated liver perfusion (OS: 9, 6–27, 4 months), or selective internal radiation therapy (OS: 18 months in monotherapy and 26 months in combination with other therapies) showed some superiority when compared to systemic therapies, such as chemotherapy (OS: 4, 6–17 months), immunotherapy (OS: 5–19, 1 month), immunosuppression (OS: 11 months), or targeted therapy (OS: 6–12 months), without being significant. Conclusions: The results of this review suggest that there are no important differences in OS when comparing the different current treatment modalities. Most of the differences found seem to be explained by the heterogenicity of the different studies and the presence of biases in their design, rather than actual extensions of patient survival.

## 1. Introduction

Uveal melanoma (UM) is the most common primary intraocular malignancy in adults. The most frequent location is the choroid, representing 80% of the total, followed by the ciliary body, 12%, and the iris, 8% [1,2]. The incidence of UM ranges from 5.3 to 10.9 cases per million inhabitants per year [3]. Risk factors for developing UM include fair skin, congenital ocular melanocytosis, melanocytoma, and BAP1-tumor predisposition syndrome [4]. Eye treatment aims to preserve the eye and useful vision and consists of various forms and combinations of phototherapy, radiotherapy, and local resection, using enucleation for the most advanced cases [5].

Despite the relatively good response of the primary UM to treatment, almost 50% of patients will develop metastatic disease. Clinically evident metastatic disease at initial presentation is detected in less than 1% of all patients [6]. However, long-term follow-up of treated patients reveals metastases in 31% of cases within 5 years, 45% within 15 years, and almost 50% within 25 years [7]. The most common primary site for the development of metastasis is the liver (89%), where dissemination through the blood vessels occurs exclusively. Due to the hematogenous spread, new blood biomarkers are being evaluated to predict and assess response to treatment. These include growth factors, microRNAs, immune markers, circulating systemic tumor cells, and beta-2-microglobulin molecules [8,9,10,11]. Recent studies highlight the importance of cytogenetic characteristics in the prognosis of UM. Thus, chromosome 3 loss is associated with a reduction in the probability of 5-year survival from 100% to 50%. In turn, chromosome 8 gain and 1 loss correlate significantly with poorer survival [12,13,14].

Therefore, improvements in primary tumor management have not translated into longer survival in patients with UM [15,16]. The 5-year survival rate among patients with the primary disease is approximately 60–70%; however, in advanced stages, with the presence of metastatic disease, the median overall survival falls to approximately 6–10 months, with only 8% of patients surviving to 2 years [13,17]. There has been considerable development in the field of treatment of metastatic UM over the past few decades, but new treatments do not appear to have demonstrated clear benefits [18,19].

To date, there is no consensus on setting the gold standard treatment, and the importance of clarifying the correct management of metastatic UM becomes apparent. In this review, we examined the effectiveness of current treatment regimens for the management of metastatic UM and their benefits on the survival of these patients.

## 2. Results

### 2.1. Conventional Chemotherapy

Most of the systemic treatments in metastatic UM have been extrapolations from experience in cutaneous melanoma. With regard to conventional chemotherapy, the most commonly used drugs have been dacarbazine, fotemustine, and temozolomide, although studies have also been conducted with more modern agents, such as docosahexaenoic acid and paclitaxel and liposomal vincristine [20]. However, unlike its cutaneous counterpart, UM tends to be characterized by chemoresistance, as shown by the average survival rates provided by this review, which range from 4.6 to 17 months [20,21,22,23,24,25,26,27,28,29,30,31,32,33,34].

In recent years, studies on temozolomide and dacarbazine with medians of OS between 5 and 13 months and progression-free interval (PFI) of up to 5.5 months have been the most consistent within this group [20,21,23,24,26,31]. The results with the combination of treosulfan and gemcitabine, on the other hand, are the most encouraging, even reaching medians of 14 months and annual survival rates of 80%, as in the Pföhler et al.’s trial [30].

Despite the large number of studies concerning conventional therapy, very few present exceptional data that deviate from the average. Among them, we found one patient alive at 5 years in the study by Leyvraz et al. [29], with fotemustine, a 57-month survival patient in the trial by Terheyden et al. [35], with gemcitabine and treosulfan, and another patient who survived 72 months in the study by Schinzari et al. [25], with a combination of cisplatin, dacarbazine, and vinblastine.

**Figure 1:**
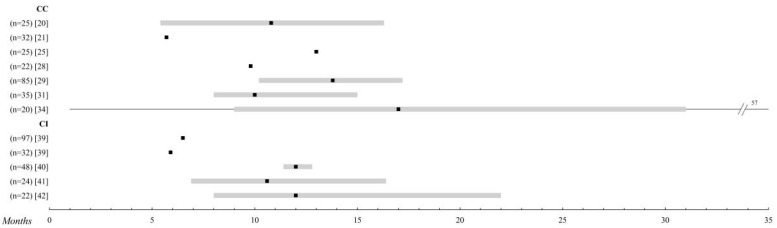
Systemic chemotherapy. Comparison of the overall survival of the different treatments in metastatic uveal melanoma (UM). Those studies with n > 20 patients were selected. The black square indicates the median, and the 95% confidence intervals are represented in a grey bar. The range is established by the black lines. The overall survival is shown up to 35 months; when it is extended, the maximum time data (in months) is added. Abbreviations: CC, conventional chemotherapy; CI, chemoimmunotherapy.

In terms of adverse effects, nausea and vomiting induced by chemotherapy represent the most frequent toxic effect, appearing in approximately 40–50% of the patients in the studies, and although they are often mild, they constitute one of the phenomena, which most deteriorate the quality of life of the oncological patient [36]. The vast majority of chemotherapy agents negatively affect the hematopoietic system [37,38], providing the most serious toxicities by affecting all cell series. Fotemustine [29] has the highest toxicity data, and dacarbazine or temozolomide [23] has the lowest (Figure 1 and Table S1).

### 2.2. Chemoimmunotherapy

Different hypotheses maintain that the immune privilege enjoyed by the eye favors the growth and development of complex tissues, thanks to different mechanisms for the suppression of immune responses. Thus, to address the difficulty of metastatic treatment, different combinations of chemo-immune therapy have been proposed [39].

It is noteworthy that in this group [35,40,41,42,43,44,45], with the exception of the study of Pyrhönen et al. [43], all treatments are specifically studied as a first-line. The overall survival data range from 3, 7 to 12 months, although the range of PFI is much wider (1, 6–12 months). A multi-center study has analyzed the efficacy of BOLD (bleomycin, vincristine, lomustine, and dacarbazine) plus recombinant interferon α-2b, a form of TIQ, due to very promising pilot reports [46,47], but ultimately the expected results could not be confirmed. Adverse effects are practically superimposed on those of isolated chemotherapy, except for complications arising from the intra-arterial catheter used in the study by Becker et al. [41] and the liver toxicity of up to 80% found by Kivelä et al. [42] (Figure 1 and Table S2).

### 2.3. Intra-Arterial Liver Chemotherapy

Intra-arterial liver chemotherapy (IAC) involves placing an infusion pump under the skin of the abdomen connected by a catheter to the liver artery. Chemotherapy agents are injected with a needle through the skin into the pump’s reservoir and are slowly and steadily released into the hepatic artery [48].

The median survival of the studies included in this review is around 15 months [49,50,51,52,53,54,55,56,57,58,59,60,61]. The multi-center trial of Leyvraz et al. [29] with fotemustine, one of the most powerful treatments in the review, is a perfect example of these results, with overall survival (OS) of 14 months and a PFI of 4.5. In this study, one patient lived exceptionally 5 years after treatment. Siegel et al. [58], also using fotemustine, reported an OS of up to 22 months, although it is not known how many of the patients included in the study received first-line therapy. On the other hand, the study by Boone et al. [49], the most recent in this group, barely reached 3 months of OS with melphalan.

**Figure 2:**
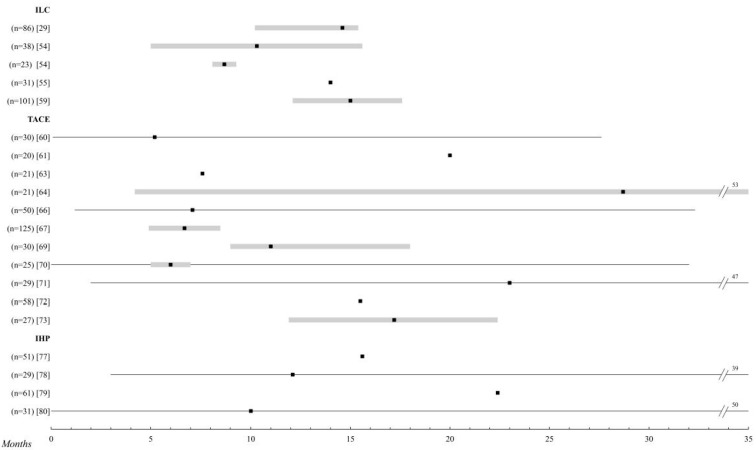
Locoregional chemotherapy. Comparison of the overall survival of the different treatments for metastatic UM. Those studies with n > 20 patients were selected. The black square indicates the median, and the 95% confidence intervals are represented in a grey bar. The range is established by the black lines. The overall survival is shown up to 35 months; when it is extended, the maximum time data (in months) is added. Abbreviations: ILC, intra-arterial liver chemotherapy; TACE, transarterial liver chemoembolization; IHP, isolated liver perfusion.

The adverse effects of this type of therapy are not striking, with percentages of myelosuppression very similar to those of the last two sections, chemotherapy and chemoimmunotherapy. Only one patient died after treatment with melphalan due to post-treatment liver failure [54] (Figure 2 and Table S3).

### 2.4. Transarterial Liver Chemoembolization

Hepatic artery chemoembolization (HAC, also called TACE (transarterial liver chemoembolization)) combines hepatic artery embolization with simultaneous infusion of concentrated doses of chemotherapy drugs. The theoretical advantages of this technique include ischemia in the metastatic area, and like other therapies aimed at the liver, achieving high pharmacological concentrations by reducing systemic toxicity [60].

The results of this group of studies are very similar to those of the IAC [26,61,62,63,64,65,66,67,68,69,70,71,72,73,74]. The average survival of the set of studies would be represented by about 10 months, with large variations between them. It should be noted that, except in two studies [66,72] (12.5%), this option has never been used as a first-line treatment, which could result in bias when interpreting the results. Similarly, the exceptional events are also very similar to the previous treatment group with three patients alive at 69, 54, and 60 months in the studies by Huppert et al. [68], Mavligit et al. [69], and Valsecchi et al. [73], respectively.

The adverse events again show a slight decrease in systemic toxicity, as in IAL, and only five associated deaths stand out in the study by Gupta et al. [67] and one due to disseminated intravascular coagulation (DIC) and multiple cerebral infarctions in the study by Carling et al. [26] (Figure 2 and Table S4).

### 2.5. Isolated Liver Perfusion

Isolated hepatic perfusion (IHP) is a surgical procedure that allows complete vascular isolation of the liver to enable the administration of high doses of chemotherapy directly to liver metastases while limiting systemic toxicity [75].

Several studies [76,77,78,79,80,81,82,83] have evaluated IHP with melphalan for liver metastases of UM with very encouraging results. Despite the fact that the OS in most studies does not differ significantly from the rest of the therapeutic groups, Ben-Shabat et al. [79] obtained a mean survival of 22.4 months. These data could be considered exceptional; however, recently, Artzner et al. [76] found in their study an OS f 27.4 months and an PFS of 11.1, thus ratifying the previous results (Figure 2 and Table S5).

### 2.6. Immunotherapy

Immunotherapy has been shown to have a large survival benefit in the treatment of metastatic skin melanoma; however, it is unclear whether this advantage translates into UM, as it is a less immunogenic tumor [84].

For this reason, it has been precisely this immunological field on which studies in recent years have focused their attention in a very noticeable way, as can be seen in the articles included in this review [21,22,50,85,86,87,88,89,90,91,92,93,94,95,96,97,98,99,100,101,102,103,104,105,106].

Ipilimumab, a monoclonal antibody to cytotoxic T-lymphocyte antigen 4 (CTLA-4), blocks the effects of this regulator and increases T-cell responses against cancer cells, thus promoting increased immune system performance. Phase III trials have shown a clear survival benefit in metastatic skin melanoma [107]. However, in UM, survival is not increased with average values of approximately 10 months [21,86,91,98].

Anti-PD1 (anti-receptor of programmed death) therapy, such as pembrolizumab or nivolumab, is also being studied, but the results are very similar to those of ipilimumab in both OS and PFI, which is about 2–3 months long [21,89,90,93,94,97,100].

The best results of this group are obtained by combining several immunotherapies, as in Kirchberg et al. [92], (Ipilimumab + Pembrolizumab), and Pelster et al. [96], (Nivolumab + Ipilimumab), with median OS of 18.4 and 19.1 months, respectively.

In this treatment group, it is remarkable how a few exceptional events are observed in certain studies. Extreme survival can be appreciated in the four alive patients at 5 years in the study by Klemen et al. [85] or the 46 months reached by a patient in the study by Bol et al. [21] with ipilimumab.

**Figure 3:**
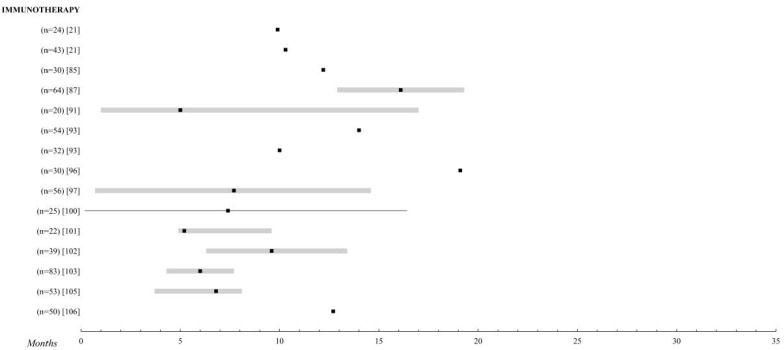
Immunotherapy. Comparison of the overall survival of the different treatments for metastatic UM. Those studies with n > 20 patients were selected. The black square indicates the median, the 95% confidence intervals are the grey bar. The range is established by the black lines. The overall survival is shown up to 35 months; when it is extended, the maximum time data (in months) is added.

The adverse effects are closely related to the hyperactivation of the immune system, with skin reactions and pseudo-flu symptoms being the most frequent in this group, which, in the vast majority of studies, are described as easily controllable. Among the most serious, hormonal alterations at the thyroid and pituitary levels and autoimmune colitis stand out, which, in the study by Rozeman et al. [86] with 10 mg of ipilimumab, affected 47% of the patients included (Figure 3 and Table S6).

### 2.7. Targeted Therapy

**Figure 4:**
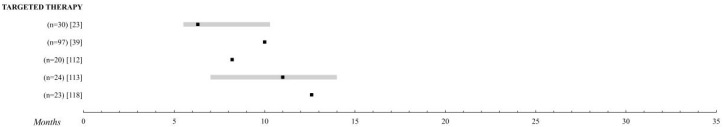
Targeted therapy. Comparison of the overall survival of the different treatments for metastatic UM. Those studies with n > 20 patients were selected. The black square indicates the median, the 95% confidence intervals are the grey bar. The range is established by the black lines. The overall survival is shown up to 35 months; when it is extended, the maximum time data (in months) is added.

Targeted therapy refers to drugs designed to interfere with a specific molecular pathway that is believed to play a critical role in tumor development or progression [108]. UM has a distinctive genetic profile that makes it an attractive candidate for the treatment with molecular target therapy. Unlike skin melanomas, BRAF mutations are extremely rare in uveal melanomas, where the vast majority show mutations in the genes GNAQ and GNA11 [109,110] that activate the mitogen-activated protein kinase (MAPK) pathway and, consequently, result in increased cell proliferation. These include drugs that can modify the pathways that regulate the cell cycle, inhibit the molecules involved in invasion and metastasis, and inhibit tumor angiogenesis [108] (Figure 4 and Table S7).

#### 2.7.1. MAPK Inhibitors

With the exception of Shah et al. [111], all the studies have focused on this path. The targeted therapies explored have been sunitinib [112], sorafenib [113,114,115], imatinib [116,117], cabozatinib [23,118], and selumetenib [40] alone or in combination with chemotherapy, reaching a median OS at 6, 3–12 months. The work of Niederkorn et al. [115] with sorafenib and fotemustine was unremarkable, providing an OS of 15.9 months and a 75% survival rate at one year, but this might be of little relevance due to the small sample size of the study—25 patients.

Promising results had initially been obtained with selumetinib, a phosphorylation inhibitor of MAPKs versus chemotherapy, but the recent SUMIT study led by Carvajal et al. [40], a phase II trial intended to confirm these results, finally found no difference between dacarbazine and dacarbazine with selumetinib with a median OS of about 10 months and a PFI of 2.8.

#### 2.7.2. Heat Shock Protein 90 Protein Inhibitor

Hsp90 (heat shock protein) is a 90 kDa chaperone that promotes the folding of other proteins, allowing them to acquire their native three-dimensional conformation and thus perform their biological function [119]. It interacts with several client proteins, including signaling kinases (RAF and AKT), growth factor receptors (MET and KIT), and cell cycle regulators [120,121]. Several studies have found overexpression of Hsp90 in both solid and hematological malignancies, and data from cell line-based experiments suggest that this overexpression can also be seen in UM [122].

Ganetespib (STA-9090) is a synthetic small molecule that binds to Hsp90 and inactivates it [123]. Preclinical data have shown that in both in vitro and in vivo systems, ganetespib exhibits potent cytotoxicity and anti-tumor activity. Taking this as a reference, Shah et al. [111] conducted a prospective, controlled clinical trial with 17 patients in which different dosages of the drug were evaluated. Unfortunately, the median OS did not exceed 8.5 months.

### 2.8. Liver Radioembolization

Liver radioembolization, also known as selective internal radiation therapy (SIRT), aims to deliver high doses of radiation to the malignant tissue while preserving the normal parenchyma, thus limiting radiation-associated injury. Yttrium-90 (^90Y^) has traditionally been used for malignant liver tumors. The small size of the ^90Y-^charged microspheres allows them to preferentially lodge in the tumor microcirculation, delivering radiation to a small circumferential area [124].

Treatment with SIRT offers a median OS at around 18 months, as first-line therapy, with very homogeneous results in the included studies [125,126,127,128,129,130,131,132]. When combined with other therapies, such as immunotherapy (ipilimumab, nivolumab, pembrolizumab, or Il-2) or immunoembolization (IE), OS rises slightly, even reaching 26 months in the study by Levey et al. [125], in which extreme values were also obtained with 95 and 120 months of survival in two patients. Values that are considerably different from the median were also found in the study by Gonsalves [127].

**Figure 5:**
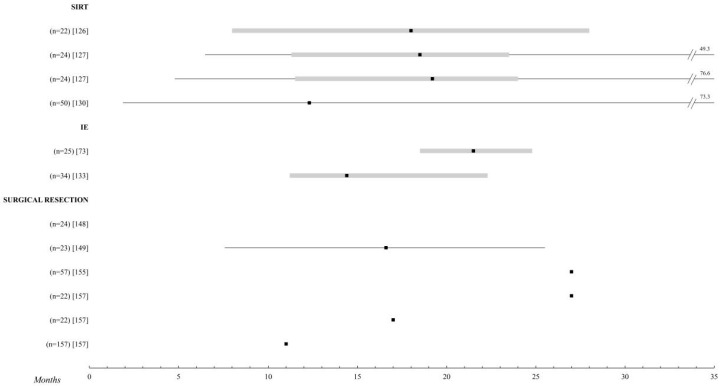
Other locoregional treatments. Comparison of the overall survival of the different treatments for metastatic UM. Those studies with n > 20 patients were selected. The black square indicates the median, the 95% confidence intervals are the grey bar. The range is established by the black lines. The overall survival is shown up to 35 months; when it is extended, the maximum time data (in months) is added. Abbreviations: SIRT, selective internal radiation therapy; IE, immunoembolization.

Treated patients do not appear to suffer significant morbidity. Post-radioembolization syndrome, characterized by fatigue, anorexia, nausea, and abdominal pain, has been the most common in this series. It is milder than post-embolization syndrome and remits easily after symptomatic treatment. Only in two patients did a gastrointestinal ulcer occurs, caused by extrahepatic deposition of the injected material, and there was only one death, in the study by Klingenstein et al. [131], due to hepatomegaly and subsequent liver failure (Figure 5 and Table S8).

### 2.9. Immunoembolization

Immunoembolization using sargramostim or granulocyte-macrophage colony-stimulating factor (GM-CSF) involves the induction of tumor-specific B and T cells to produce local inflammation and regression of metastatic lesions [133].

The only two studies evaluating this therapy belong to the same center—a phase I trial and its subsequent phase II [73,133]. In the latter, they have obtained a median OS of 21.5 months and compared immunoembolization with soft transarterial chemoembolization, finding that overall survival is higher in the IE group in patients with more than 20% liver involvement, but they have observed no difference in those with less liver involvement. Extreme survival can be found in both studies [77,133], represented by 50 and 40.8 months of two patients, respectively (Figure 5 and Table S9).

### 2.10. Immunosuppression

The signaling of mTOR (the target of rapamycin in mammalian cells) is unregulated in a wide range of human cancers [134]. In UM, 60% of metastatic tumors show a loss of tumor suppressors that inhibit this pathway [135]. However, clinical resistance to mTOR monotherapy is common, and combined therapeutic strategies are needed for sustained clinical benefit. A potential mechanism of resistance to mTOR inhibition is the rebound activation of the insulin-like growth factor receptor 1 (IGF1R) pathway signaling [136]. In UM, the expression of tumor IGF1R has been associated with disease progression, and the in-vitro inhibition of IGF1R results in tumor regression of UM [137,138].

Thus, the study by Shoushtari et al. [139] investigated whether a combined inhibition of IGF1R and mTOR could provide a clinical benefit in UM through a single-arm phase II trial of combined inhibition of mTOR and IGF1R with everolimus and pasireotide in patients with metastatic UM. The clinical benefit shown was very limited, with an OS of 11 months and the need to reduce the treatment dose to avoid adverse effects (Table S10).

### 2.11. Liver Thermotherapy

Other local ablative treatment techniques, such as CT-guided multi-probe stereotactic radiofrequency ablation (SRFA) or percutaneous magnetic resonance imaging (MRI)-guided laser-induced interstitial thermotherapy (LITT), have also been investigated for metastatic UM. The survival results are spectacular, reaching 38 months of the median OS with SRFA [140] and 33.6 months with LITT [141], making them an attractive alternative enhanced by minimal adverse effects. However, the scarcity of studies and their small sample size make it clear that new work is needed to confirm these theories (Table S11).

### 2.12. Dendritic Cell Vaccine

Dendritic cell vaccines loaded with melanoma antigens have long been investigated for the treatment of cutaneous melanoma. A phase II study has already found favorable results at the preventive level when evaluating their use in patients with primary UM and chromosome 3 monosomy, comparing their survival results with historical controls [142].

In the study included in this review, Bol et al. [143] found a median OS of 19.2 months for metastatic UM. In addition, it reported a 50% survival of patients at two years, with one patient alive at 84 months, and with minimal side effects, concluding that this is a feasible and safe option, although more studies are needed to corroborate these results (Table S12).

### 2.13. Oncolytic Adenovirus Icovir-5

Oncolytic viruses represent a unique type of agent that combines self-amplifying, lytic, and immunostimulating properties against tumors. Therapy based on them relies on the ability of the virus to infect and selectively replicate in tumor cells, leading to oncolysis and the release of new viruses, which lead to local and bloodstream spread by inducing an immune response against the tumor [144,145].

The preclinical efficacy of a single intravenous administration of the oncolytic adenovirus ICOVIR5, a type 5 adenovirus that responds to the pRB pathway, commonly unregulated in tumors, led Garcia et al. [146] to use this virus in a phase I trial of metastatic UM. The results of 8.9 months of median OS in six patients, treated with a single infusion of up to 1E13 viral particles, showed that ICOVIR5 could achieve melanoma metastasis with a single intravenous administration but could not induce tumor regression (Table S13).

### 2.14. Surgical Resection

The longest average survival times in patients with metastatic UM are observed in this group, with median OS tending to reach 2 years. This requires patients to meet a series of conditions that allow complete resection of the solitary metastasis, either in the liver [147,148,149,150] or in other locations. This explains why when patients are eligible, surgical resection becomes the first treatment option, as can be seen in all the included studies [57,148,149,150,151,152,153,154,155,156,157] with the exception of Aoyama et al. [150].

In the comparative case series of Mariani et al. [155], it is shown that the combination of surgery with RFA(radiofrequency ablatio) has an overall survival and progression-free interval similar to surgery alone. The combination of surgery with chemotherapy has also been investigated with the aim of improving results in studies by Kodjikian et al. [151], Rivoire et al. [152], and Salmon et al. [57], where it is not clear that it results in an actual prolongation of survival compared to isolated metastatic resection, and if it does, it would be a very subtle benefit.

As for exceptional events, extreme survivors beyond the age of 5 are relatively frequent in this group. Among these, the study by Servois et al. [153], combining surgery with AFRS, stands out, in which 10 patients (71.4%) reached 5 years of survival (Figure 5 and Table S14).

## 3. Discussion

In the present systematic review, a hypothesis has been made about the effectiveness of current treatment schemes for metastatic UM. We observe that only six of the 110 studies included have been randomized, which already poses a methodological problem in the remaining 104, in which the presence of confounding bias could exist. Most articles (59.1%) are not based on experimental studies, but on well-designed observational studies.

In this review, OS has been chosen as the final point because it is considered a more significant result and of greater relevance to the patient because the stabilization of the disease and even slower progression are important aspects [158]. Furthermore, it represents data that tend to be available frequently at an individual level and less subject to interpretation.

However, there are also limitations, in that there are differences in study methodology that can influence the final results, and these must be taken into account when evaluating them. Most of the included studies are retrospective and have a small sample size. The high level of heterogenicity becomes an important limitation, as a result of publication and selection biases and the variability of the different cases. Some metastases are detected by surveillance and others after the development of symptoms; some patients have not received treatment, and others have received intense pretreatment, so it seems necessary to reflect the percentage of patients who underwent treatment as a first-line. Another bias that has been attempted to be taken into account in analyzing the results is metastasis resection, which, even when partial, can prolong the patient’s survival [154,159]. However, other sources of bias, including functional status, metastatic locations and burden, and liver function are reported variably, and not at the individual patient level, and could not be reflected.

When evaluating the various treatment options together, there does not currently appear to be an effective treatment for advanced-stage UM. The results of this review suggest that although regional therapies appear to show some superiority compared to systemic therapies, most of the differences found are explained by the heterogenicity of the different studies and the presence of biases in their designs, rather than actual prolongations of patient survival.

As described by Rietschel et al., in the present review, exceptional outcomes are observed, especially in the surgical resection and locoregional treatment groups [160]. This finding could be due to a bias toward worse survival of patients with the more disseminated disease who are less likely to be treated with localized therapy. Within the group of immunotherapy, it should be highlighted the work of Klemen et al., where 20% of patients reach 5 years of survival; the authors have attributed this result to those patients that have received anti-CTLA-4 and anti-PD-1, either sequentially or in combination. Although they cannot rule out that it is due to the selection of the patients or to a reduced number of the samples [85].

The different methodologies employed in the original publications have posed a challenge to this review. Occasionally, the median OS is not provided, or the Kaplan–Meier graphs lack risk tables and censored events, introducing potential biases for data analysis. These problems are in agreement with those reported in the meta-analysis by Rantala et al. [161], who have already proposed a series of guidelines so that future studies can report results on the treatment of metastatic UM in a more systematic way and allow more consistent results to be obtained. The small sample size of the included patients and the absence of randomization makes direct comparability and interpretation difficult. For this reason, we believe that an effort is needed on the part of researchers to carry out protocolized studies with a common system that allows direct comparability to reach consistent conclusions about the benefit of different treatments in the survival of patients with metastatic uveal melanoma and to explore new treatment routes that can be used effectively in this type of patient.

The UM has been differentiated into different molecular subsets, which differ in their genetic aberrations, pattern of methylation, gene expression profile (GEP), and metabolomic and immunological characteristics [162,163,164]. UMs with favorable prognosis are characterized by disomy 3, a class 1 GEP, and two copies of chromosome 8q. In contrast, prognostically unfavorable UMs are highly lethal; these UMs show monosomy 3, a GEP class 2, BAP1 inactivation, multiple copies of chromosome 8q [165,166]. Abdel-Rahman et al. have suggested that molecular genetic alterations of the tumor, in particular, the lack of monosomy 3, are associated with such prolonged survival [167]. In our review, very few studies provide information related to the genetic characteristics of the sample [93,94,97,118,139,143,153], and in those that are studied statistically associated with survival, no differences have been found [23,40,89]. Tumor molecular profiles are becoming increasingly important in the UM, and we believe that it would be important to apply this new knowledge to future treatment trials in these patients with metastasis.

## 4. Methods

The search strategy carried out on 13 April 2020 in PubMed, without applying language restrictions and within the time interval from 1 January 1980 to 13 April 2020, was as follows: (uveal melanoma OR choroidal melanoma OR ciliary body melanoma OR ciliochoroidal melanoma OR iridociliary melanoma OR iris melanoma OR intraocular melanoma OR ocular melanoma) AND (metast * OR stage IV) AND (treatment) AND (‘1980/01/01′ [PDAT]: ‘2020/04/13′ [PDAT]) with 2098 references. Those articles that were reviews or case reports were excluded. Besides, those based on animal models, laboratory investigations, imaging studies, primary tumor or local recurrence, prognosis, staging, or quality of life were also excluded. Two authors (CV and MB) separately reviewed and, based on the selection criteria, decided the suitability of the articles for inclusion. The disagreement was resolved through discussions with a third reviewer (AP).

In this study, the main objective was effectiveness in terms of overall survival (OS), understood as the time elapsed until the event of interest, death in this case, and whose exact definition has been obtained according to the specifications of each article. To this end, it is necessary to refer to the Response Evaluation Criteria in Solid Tumors (RECIST), which consists of a standardized method to measure the way in which an oncological patient responds to treatment. These standards make it possible to determine whether tumors are shrinking, staying the same, or getting bigger [168]. Based on this, if the articles included in the analysis referred to the RECIST criteria, it was assumed that the measure of OS went from the time of initiation of treatment to study to censorship or death. If no reference was made to these criteria, and multiple definitions were provided, the one corresponding to the Kaplan–Meier method was chosen.

**Figure 6:**
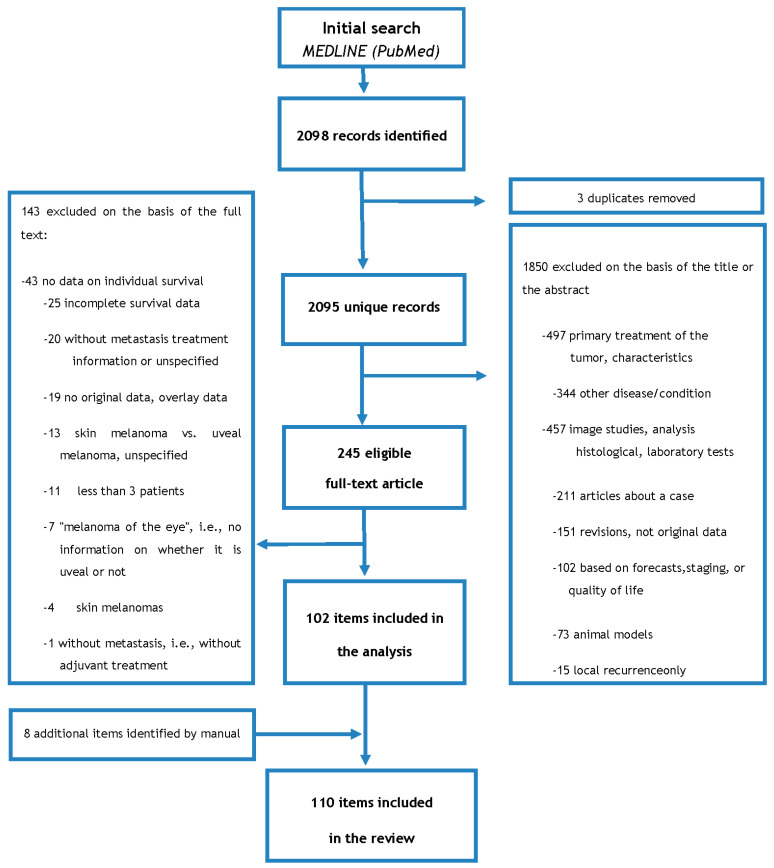
Article selection flowchart.

Preferred reporting items for systematic reviews (PRISMA) guidelines were followed (Figure 6) [169], reducing the eligible studies through different selection strategies. After this, a text review of the 245 selected articles was carried out, obtaining 102 articles that met all the required characteristics. At this point, studies that included a primary cutaneous or mucosal melanoma were discarded unless patients with UM were studied independently. Finally, we performed a manual search of the reference lists of the articles themselves and identified a further 8 articles, obtaining a final result of 110 articles included in this review. In this systematic review, we first extracted and tabulated data corresponding to the author, year of publication, treatment, study design, number of patients, first-line treatments and previous surgeries, OS with its definition, adverse effects of each treatment, and median OS. Exceptional outcomes were defined as patients who survived more than 4 years with metastasis [160]. These data can be seen in Supplementary Data.

To analyze and evaluate the quality of each of the selected articles, the levels of scientific evidence and recommendations of the Agency for Healthcare Research and Quality were used [170], obtaining the results shown in Table 1. Of the 110 articles included in the review, 47 (42.73%) were prospective studies, and 63 (57.27%) were retrospective studies. In reference to OS, the starting point was reported from the diagnosis of metastasis in 10 (9.09%), enrolment in the study in 8 (7.27%), initiation of treatment in 84 (76.36%), and was not defined in 8 (7.27%) publications. In addition, multiple definitions were adopted in 11 articles. Regarding conflicts of interest, 31 (28.18%) studies were fully or partially funded by industry, with authors reporting a conflict of interest, or both. In 54 (49.1%) studies, the industry was not involved, and in 25 (22.72%), this information was not available.

## 5. Conclusions

There do not appear to be any notable differences in OS when comparing the different current metastatic UM treatment modalities. Most of the differences found seem to be explained by the heterogeneity of the different studies and the presence of biases in their design, rather than by the actual extensions of patient survival.

According to data from the National Cancer Institute, around 20 clinical trials are being conducted worldwide with the aim of finding new answers in the treatment of metastatic UM. These studies exemplify where the future of treatment of metastatic UM is headed. The new research advocates immunotherapy as the most promising treatment for the future and looks to these drugs to find real improvements in survival for these patients.

## Figures and Tables

**Table 1:** Classification of articles according to the level of evidence (Agency for Healthcare Research and Quality).

Level	n	%
Ia	0	0
Ib	6	5.5
IIa	23	20.9
IIb	16	14.5
III	65	59.1
IV	0	0
TOTAL	110	100%

## Supplementary Materials

The following are available online at https://www.mdpi.com/2072-6694/12/9/2557/s1. Table S1. Included studies of conventional chemotherapy; Table S2. Included studies of chemoimmunotherapy; Table S3. Included studies of intra-arterial liver chemotherapy (ILC); Table S4. Included studies of transarterial liver chemoembolization (TACE); Table S5. Included liver perfusion isolation studies (IHP); Table S6. Included studies of immunotherapy; Table S7. Included targeted therapy studies; Table S8. Included liver radioembolization studies; Table S9. Included immunoembolization studies; Table S10. Included studies of immunosuppression; Table S11. Included studies of thermotherapy directed at the liver; Table S12. Included dendritic cell vaccine studies; Table S13. Included studies of CIOVIR-5 oncolytic adenovirus; Table S14. Included studies of surgical resection.

## References

[1] Strickland D., Lee J.A. (1981). Melanomas of eye: Stability of rates. Am. J. Epidemiol..

[2] Kivela T. (2009). The epidemiological challenge of the most frequent eye cancer: Retinoblastoma, an issue of birth and death. Br. J. Ophthalmol..

[3] Scotto J., Fraumeni J.F., Lee J.A. (1976). Melanomas of the eye and other noncutaneous sites: Epidemiologic aspects. J. Natl. Cancer Inst..

[4] Nayman T., Bostan C., Logan P., Burnier M.N. (2017). Uveal Melanoma Risk Factors: A Systematic Review of Meta-Analyses. Curr. Eye Res..

[5] Damato B. (2006). Treatment of primary intraocular melanoma. Expert Rev. Anticancer Ther..

[6] Diener-West M., Reynolds S.M., Agugliaro D.J., Caldwell R., Cumming K., Earle J.D., Green D.L., Hawkins B.S., Hayman J., Jaiyesimi I. (2004). Screening for metastasis from choroidal melanoma: The Collaborative Ocular Melanoma Study Group Report 23. J. Clin. Oncol..

[7] Kujala E., Makitie T., Kivela T. (2003). Very long-term prognosis of patients with malignant uveal melanoma. Investig. Ophthalmol. Vis. Sci..

[8] Bande M.F., Santiago M., Mera P., Piulats J.M., Blanco M.J., Rodriguez-Alvarez M.X., Capeans C., Pineiro A., Pardo M. (2015). ME20-S as a Potential Biomarker for the Evaluation of Uveal Melanoma. Investig. Ophthalmol. Vis. Sci..

[9] Bande Rodriguez M.F., Fernandez Marta B., Lago Baameiro N., Santiago-Varela M., Silva-Rodriguez P., Blanco-Teijeiro M.J., Pardo Perez M., Pineiro Ces A. (2020). Blood Biomarkers of Uveal Melanoma: Current Perspectives. Clin. Ophthalmol..

[10] Barak V., Kaiserman I., Frenkel S., Hendler K., Kalickman I., Pe’er J. (2011). The dynamics of serum tumor markers in predicting metastatic uveal melanoma (part 1). Anticancer Res..

[11] Pardo M., Dwek R.A., Zitzmann N. (2007). Proteomics in uveal melanoma research: Opportunities and challenges in biomarker discovery. Expert Rev. Proteomics.

[12] Dogrusoz M., Jager M.J. (2018). Genetic prognostication in uveal melanoma. Acta Ophthalmol..

[13] Damato B., Eleuteri A., Taktak A.F., Coupland S.E. (2011). Estimating prognosis for survival after treatment of choroidal melanoma. Prog. Retin. Eye Res..

[14] Dogrusoz M., Bagger M., van Duinen S.G., Kroes W.G., Ruivenkamp C.A., Bohringer S., Andersen K.K., Luyten G.P., Kiilgaard J.F., Jager M.J. (2017). The Prognostic Value of AJCC Staging in Uveal Melanoma Is Enhanced by Adding Chromosome 3 and 8q Status. Investig. Ophthalmol. Vis. Sci..

[15] Singh A.D., Turell M.E., Topham A.K. (2011). Uveal melanoma: Trends in incidence, treatment, and survival. Ophthalmology.

[16] Abrahamsson M. (1983). Malignant melanoma of the choroid and the ciliary body 1956-1975 in Halland and Gothenburg. Incidence, histopathology and prognosis. Acta Ophthalmol..

[17] Krantz B.A., Dave N., Komatsubara K.M., Marr B.P., Carvajal R.D. (2017). Uveal melanoma: Epidemiology, etiology, and treatment of primary disease. Clin. Ophthalmol..

[18] Augsburger J.J., Correa Z.M., Shaikh A.H. (2009). Effectiveness of treatments for metastatic uveal melanoma. Am. J. Ophthalmol..

[19] Buder K., Gesierich A., Gelbrich G., Goebeler M. (2013). Systemic treatment of metastatic uveal melanoma: Review of literature and future perspectives. Cancer Med..

[20] Pons F., Plana M., Caminal J.M., Pera J., Fernandes I., Perez J., Garcia-Del-Muro X., Marcoval J., Penin R., Fabra A. (2011). Metastatic uveal melanoma: Is there a role for conventional chemotherapy?—A single center study based on 58 patients. Melanoma Res..

[21] Bol K.F., Ellebaek E., Hoejberg L., Bagger M.M., Larsen M.S., Klausen T.W., Kohler U.H., Schmidt H., Bastholt L., Kiilgaard J.F. (2019). Real-World Impact of Immune Checkpoint Inhibitors in Metastatic Uveal Melanoma. Cancers.

[22] Xu L.T., Funchain P.F., Bena J.F., Li M., Tarhini A., Berber E., Singh A.D. (2019). Uveal Melanoma Metastatic to the Liver: Treatment Trends and Outcomes. Ocul. Oncol. Pathol..

[23] Luke J.J., Olson D.J., Allred J.B., Strand C.A., Bao R., Zha Y., Carll T., Labadie B.W., Bastos B.R., Butler M.O. (2020). Randomized Phase II Trial and Tumor Mutational Spectrum Analysis from Cabozantinib versus Chemotherapy in Metastatic Uveal Melanoma (Alliance A091201). Clin. Cancer Res..

[24] Tulokas S., Maenpaa H., Peltola E., Kivela T., Vihinen P., Virta A., Makela S., Kallio R., Hernberg M. (2018). Selective internal radiation therapy (SIRT) as treatment for hepatic metastases of uveal melanoma: A Finnish nation-wide retrospective experience. Acta Oncol..

[25] Schinzari G., Rossi E., Cassano A., Dadduzio V., Quirino M., Pagliara M., Blasi M.A., Barone C. (2017). Cisplatin, dacarbazine and vinblastine as first line chemotherapy for liver metastatic uveal melanoma in the era of immunotherapy: A single institution phase II study. Melanoma Res..

[26] Carling U., Dorenberg E.J., Haugvik S.P., Eide N.A., Berntzen D.T., Edwin B., Dueland S., Rosok B. (2015). Transarterial Chemoembolization of Liver Metastases from Uveal Melanoma Using Irinotecan-Loaded Beads: Treatment Response and Complications. Cardiovasc. Interv. Radiol..

[27] Corrie P.G., Shaw J., Spanswick V.J., Sehmbi R., Jonson A., Mayer A., Bulusu R., Hartley J.A., Cree I.A. (2005). Phase I trial combining gemcitabine and treosulfan in advanced cutaneous and uveal melanoma patients. Br. J. Cancer.

[28] Homsi J., Bedikian A.Y., Papadopoulos N.E., Kim K.B., Hwu W.J., Mahoney S.L., Hwu P. (2010). Phase 2 open-label study of weekly docosahexaenoic acid-paclitaxel in patients with metastatic uveal melanoma. Melanoma Res..

[29] Leyvraz S., Piperno-Neumann S., Suciu S., Baurain J.F., Zdzienicki M., Testori A., Marshall E., Scheulen M., Jouary T., Negrier S. (2014). Hepatic intra-arterial versus intravenous fotemustine in patients with liver metastases from uveal melanoma (EORTC 18021): A multicentric randomized trial. Ann. Oncol..

[30] Pfohler C., Cree I.A., Ugurel S., Kuwert C., Haass N., Neuber K., Hengge U., Corrie P.G., Zutt M., Tilgen W. (2003). Treosulfan and gemcitabine in metastatic uveal melanoma patients: Results of a multicenter feasibility study. Anticancer Drugs.

[31] Piperno-Neumann S., Diallo A., Etienne-Grimaldi M.C., Bidard F.C., Rodrigues M., Plancher C., Mariani P., Cassoux N., Decaudin D., Asselain B. (2016). Phase II Trial of Bevacizumab in Combination With Temozolomide as First-Line Treatment in Patients With Metastatic Uveal Melanoma. Oncologist.

[32] Schmittel A., Scheulen M.E., Bechrakis N.E., Strumberg D., Baumgart J., Bornfeld N., Foerster M.H., Thiel E., Keilholz U. (2005). Phase II trial of cisplatin, gemcitabine and treosulfan in patients with metastatic uveal melanoma. Melanoma Res..

[33] Schmittel A., Schuster R., Bechrakis N.E., Siehl J.M., Foerster M.H., Thiel E., Keilholz U. (2005). A two-cohort phase II clinical trial of gemcitabine plus treosulfan in patients with metastatic uveal melanoma. Melanoma Res..

[34] Terheyden P., Brocker E.B., Becker J.C. (2004). Clinical evaluation of in vitro chemosensitivity testing: The example of uveal melanoma. J. Cancer Res. Clin. Oncol..

[35] Terheyden P., Kampgen E., Runger T.M., Brocker E.B., Becker J.C. (1998). Immunochemotherapy of metastatic uveal melanoma with interferon alfa-2b, interleukin-2 and fotemustine. Case reports and review of the literature. Hautarzt.

[36] Andrews P.L., Rapeport W.G., Sanger G.J. (1988). Neuropharmacology of emesis induced by anti-cancer therapy. Trends Pharmacol. Sci..

[37] Pedersen-Bjergaard J., Rowley J.D. (1994). The balanced and the unbalanced chromosome aberrations of acute myeloid leukemia may develop in different ways and may contribute differently to malignant transformation. Blood.

[38] Pedersen-Bjergaard J., Daugaard G., Hansen S.W., Philip P., Larsen S.O., Rorth M. (1991). Increased risk of myelodysplasia and leukaemia after etoposide, cisplatin, and bleomycin for germ-cell tumours. Lancet.

[39] Ijland S.A., Jager M.J., Heijdra B.M., Westphal J.R., Peek R. (1999). Expression of angiogenic and immunosuppressive factors by uveal melanoma cell lines. Melanoma Res..

[40] Carvajal R.D., Piperno-Neumann S., Kapiteijn E., Chapman P.B., Frank S., Joshua A.M., Piulats J.M., Wolter P., Cocquyt V., Chmielowski B. (2018). Selumetinib in Combination With Dacarbazine in Patients With Metastatic Uveal Melanoma: A Phase III, Multicenter, Randomized Trial (SUMIT). J. Clin. Oncol..

[41] Becker J.C., Terheyden P., Kampgen E., Wagner S., Neumann C., Schadendorf D., Steinmann A., Wittenberg G., Lieb W., Brocker E.B. (2002). Treatment of disseminated ocular melanoma with sequential fotemustine, interferon alpha, and interleukin 2. Br. J. Cancer.

[42] Kivela T., Suciu S., Hansson J., Kruit W.H., Vuoristo M.S., Kloke O., Gore M., Hahka-Kemppinen M., Parvinen L.M., Kumpulainen E. (2003). Bleomycin, vincristine, lomustine and dacarbazine (BOLD) in combination with recombinant interferon alpha-2b for metastatic uveal melanoma. Eur. J. Cancer.

[43] Pyrhonen S., Hahka-Kemppinen M., Muhonen T., Nikkanen V., Eskelin S., Summanen P., Tarkkanen A., Kivela T. (2002). Chemoimmunotherapy with bleomycin, vincristine, lomustine, dacarbazine (BOLD), and human leukocyte interferon for metastatic uveal melanoma. Cancer.

[44] Solti M., Berd D., Mastrangelo M.J., Sato T. (2007). A pilot study of low-dose thalidomide and interferon alpha-2b in patients with metastatic melanoma who failed prior treatment. Melanoma Res..

[45] Vihinen P.P., Hernberg M., Vuoristo M.S., Tyynela K., Laukka M., Lundin J., Ivaska J., Pyrhonen S. (2010). A phase II trial of bevacizumab with dacarbazine and daily low-dose interferon-alpha2a as first line treatment in metastatic melanoma. Melanoma Res..

[46] Nathan F.E., Berd D., Sato T., Shield J.A., Shields C.L., De Potter P., Mastrangelo M.J. (1997). BOLD+interferon in the treatment of metastatic uveal melanoma: First report of active systemic therapy. J. Exp. Clin. Cancer Res. CR.

[47] Pyrhonen S. (1998). The treatment of metastatic uveal melanoma. Eur. J. Cancer.

[48] Feldman E.D., Pingpank J.F., Alexander H.R. (2004). Regional treatment options for patients with ocular melanoma metastatic to the liver. Ann. Surg. Oncol..

[49] Boone B.A., Perkins S., Bandi R., Santos E., McCluskey K., Bartlett D.L., Pingpank J.F. (2018). Hepatic artery infusion of melphalan in patients with liver metastases from ocular melanoma. J. Surg. Oncol..

[50] Itchins M., Ascierto P.A., Menzies A.M., Oatley M., Lo S., Douraghi-Zadeh D., Harrington T., Maher R., Grimaldi A.M., Guminski A. (2017). A multireferral centre retrospective cohort analysis on the experience in treatment of metastatic uveal melanoma and utilization of sequential liver-directed treatment and immunotherapy. Melanoma Res..

[51] Cantore M., Fiorentini G., Aitini E., Davitti B., Cavazzini G., Rabbi C., Lusenti A., Bertani M., Morandi C., Benedini V. (1994). Intra-arterial hepatic carboplatin-based chemotherapy for ocular melanoma metastatic to the liver. Report of a phase II study. Tumori J..

[52] Egerer G., Lehnert T., Max R., Naeher H., Keilholz U., Ho A.D. (2001). Pilot study of hepatic intraarterial fotemustine chemotherapy for liver metastases from uveal melanoma: A single-center experience with seven patients. Int. J. Clin. Oncol..

[53] Farolfi A., Ridolfi L., Guidoboni M., Milandri C., Calzolari F., Scarpi E., Amadori D., Ridolfi R. (2011). Liver metastases from melanoma: Hepatic intra-arterial chemotherapy. A retrospective study. J. Chemother..

[54] Heusner T.A., Antoch G., Wittkowski-Sterczewski A., Ladd S.C., Forsting M., Verhagen R., Scheulen M. (2011). Transarterial hepatic chemoperfusion of uveal melanoma metastases: Survival and response to treatment. Rofo.

[55] Leyvraz S., Spataro V., Bauer J., Pampallona S., Salmon R., Dorval T., Meuli R., Gillet M., Lejeune F., Zografos L. (1997). Treatment of ocular melanoma metastatic to the liver by hepatic arterial chemotherapy. J. Clin. Oncol..

[56] Melichar B., Voboril Z., Lojik M., Krajina A. (2009). Liver metastases from uveal melanoma: Clinical experience of hepatic arterial infusion of cisplatin, vinblastine and dacarbazine. Hepatogastroenterology.

[57] Salmon R.J., Levy C., Plancher C., Dorval T., Desjardins L., Leyvraz S., Pouillart P., Schlienger P., Servois V., Asselain B. (1998). Treatment of liver metastases from uveal melanoma by combined surgery-chemotherapy. Eur. J. Surg. Oncol..

[58] Siegel R., Hauschild A., Kettelhack C., Kahler K.C., Bembenek A., Schlag P.M. (2007). Hepatic arterial Fotemustine chemotherapy in patients with liver metastases from cutaneous melanoma is as effective as in ocular melanoma. Eur. J. Surg. Oncol..

[59] Peters S., Voelter V., Zografos L., Pampallona S., Popescu R., Gillet M., Bosshard W., Fiorentini G., Lotem M., Weitzen R. (2006). Intra-arterial hepatic fotemustine for the treatment of liver metastases from uveal melanoma: Experience in 101 patients. Ann. Oncol..

[60] Patel K., Sullivan K., Berd D., Mastrangelo M.J., Shields C.L., Shields J.A., Sato T. (2005). Chemoembolization of the hepatic artery with BCNU for metastatic uveal melanoma: Results of a phase II study. Melanoma Res..

[61] Farshid P., Darvishi A., Naguib N., Bazrafshan B., Paul J., Mbalisike E., Vogl T.J. (2013). Repetitive chemoembolization of hypovascular liver metastases from the most common primary sites. Future Oncol..

[62] Agarwala S.S., Panikkar R., Kirkwood J.M. (2004). Phase I/II randomized trial of intrahepatic arterial infusion chemotherapy with cisplatin and chemoembolization with cisplatin and polyvinyl sponge in patients with ocular melanoma metastatic to the liver. Melanoma Res..

[63] Dayani P.N., Gould J.E., Brown D.B., Sharma K.V., Linette G.P., Harbour J.W. (2009). Hepatic metastasis from uveal melanoma: Angiographic pattern predictive of survival after hepatic arterial chemoembolization. Arch. Ophthalmol..

[64] Edelhauser G., Schicher N., Berzaczy D., Beitzke D., Hoeller C., Lammer J., Funovics M. (2012). Fotemustine chemoembolization of hepatic metastases from uveal melanoma: A retrospective single-center analysis. AJR Am. J. Roentgenol..

[65] Fiorentini G., Aliberti C., Del Conte A., Tilli M., Rossi S., Ballardini P., Turrisi G., Benea G. (2009). Intra-arterial hepatic chemoembolization (TACE) of liver metastases from ocular melanoma with slow-release irinotecan-eluting beads. Early results of a phase II clinical study. In Vivo.

[66] Gonsalves C.F., Eschelman D.J., Thornburg B., Frangos A., Sato T. (2015). Uveal Melanoma Metastatic to the Liver: Chemoembolization With 1,3-Bis-(2-Chloroethyl)-1-Nitrosourea. AJR Am. J. Roentgenol..

[67] Gupta S., Bedikian A.Y., Ahrar J., Ensor J., Ahrar K., Madoff D.C., Wallace M.J., Murthy R., Tam A., Hwu P. (2010). Hepatic artery chemoembolization in patients with ocular melanoma metastatic to the liver: Response, survival, and prognostic factors. Am. J. Clin. Oncol..

[68] Huppert P.E., Fierlbeck G., Pereira P., Schanz S., Duda S.H., Wietholtz H., Rozeik C., Claussen C.D. (2010). Transarterial chemoembolization of liver metastases in patients with uveal melanoma. Eur. J. Radiol..

[69] Mavligit G.M., Charnsangavej C., Carrasco C.H., Patt Y.Z., Benjamin R.S., Wallace S. (1988). Regression of ocular melanoma metastatic to the liver after hepatic arterial chemoembolization with cisplatin and polyvinyl sponge. JAMA.

[70] Schuster R., Lindner M., Wacker F., Krossin M., Bechrakis N., Foerster M.H., Thiel E., Keilholz U., Schmittel A. (2010). Transarterial chemoembolization of liver metastases from uveal melanoma after failure of systemic therapy: Toxicity and outcome. Melanoma Res..

[71] Shibayama Y., Namikawa K., Sone M., Takahashi A., Tsutsumida A., Sugawara S., Arai Y., Aihara Y., Suzuki S., Nakayama J. (2017). Efficacy and toxicity of transarterial chemoembolization therapy using cisplatin and gelatin sponge in patients with liver metastases from uveal melanoma in an Asian population. Int. J. Clin. Oncol..

[72] Valpione S., Aliberti C., Parrozzani R., Bazzi M., Pigozzo J., Midena E., Pilati P., Campana L.G., Chiarion-Sileni V. (2015). A retrospective analysis of 141 patients with liver metastases from uveal melanoma: A two-cohort study comparing transarterial chemoembolization with CPT-11 charged microbeads and historical treatments. Melanoma Res..

[73] Valsecchi M.E., Terai M., Eschelman D.J., Gonsalves C.F., Chervoneva I., Shields J.A., Shields C.L., Yamamoto A., Sullivan K.L., Laudadio M. (2015). Double-blinded, randomized phase II study using embolization with or without granulocyte-macrophage colony-stimulating factor in uveal melanoma with hepatic metastases. J. Vasc. Interv. Radiol. JVIR.

[74] Vogl T., Eichler K., Zangos S., Herzog C., Hammerstingl R., Balzer J., Gholami A. (2007). Preliminary experience with transarterial chemoembolization (TACE) in liver metastases of uveal malignant melanoma: Local tumor control and survival. J. Cancer Res. Clin. Oncol..

[75] Boone B.A., Bartlett D.L., Zureikat A.H. (2012). Isolated hepatic perfusion for the treatment of liver metastases. Curr. Probl. Cancer.

[76] Artzner C., Mossakowski O., Hefferman G., Grosse U., Hoffmann R., Forschner A., Eigentler T., Syha R., Grozinger G. (2019). Chemosaturation with percutaneous hepatic perfusion of melphalan for liver-dominant metastatic uveal melanoma: A single center experience. Cancer Imaging.

[77] Karydis I., Gangi A., Wheater M.J., Choi J., Wilson I., Thomas K., Pearce N., Takhar A., Gupta S., Hardman D. (2018). Percutaneous hepatic perfusion with melphalan in uveal melanoma: A safe and effective treatment modality in an orphan disease. J. Surg. Oncol..

[78] Alexander H.R., Libutti S.K., Pingpank J.F., Steinberg S.M., Bartlett D.L., Helsabeck C., Beresneva T. (2003). Hyperthermic isolated hepatic perfusion using melphalan for patients with ocular melanoma metastatic to liver. Clin. Cancer Res..

[79] Ben-Shabat I., Belgrano V., Ny L., Nilsson J., Lindner P., Olofsson Bagge R. (2016). Long-Term Follow-Up Evaluation of 68 Patients with Uveal Melanoma Liver Metastases Treated with Isolated Hepatic Perfusion. Ann. Surg. Oncol..

[80] de Leede E.M., Burgmans M.C., Kapiteijn E., Luyten G.P., Jager M.J., Tijl F.G., Hartgrink H.H., Grunhagen D.J., Rothbarth J., van de Velde C.J. (2016). Isolated (hypoxic) hepatic perfusion with high-dose chemotherapy in patients with unresectable liver metastases of uveal melanoma: Results from two experienced centres. Melanoma Res..

[81] Forster M.R., Rashid O.M., Perez M.C., Choi J., Chaudhry T., Zager J.S. (2014). Chemosaturation with percutaneous hepatic perfusion for unresectable metastatic melanoma or sarcoma to the liver: A single institution experience. J. Surg. Oncol..

[82] van Iersel L.B., de Leede E.M., Vahrmeijer A.L., Tijl F.G., den Hartigh J., Kuppen P.J., Hartgrink H.H., Gelderblom H., Nortier J.W., Tollenaar R.A. (2014). Isolated hepatic perfusion with oxaliplatin combined with 100 mg melphalan in patients with metastases confined to the liver: A phase I study. Eur. J. Surg. Oncol..

[83] Vogl T.J., Koch S.A., Lotz G., Gebauer B., Willinek W., Engelke C., Bruning R., Zeile M., Wacker F., Vogel A. (2017). Percutaneous Isolated Hepatic Perfusion as a Treatment for Isolated Hepatic Metastases of Uveal Melanoma: Patient Outcome and Safety in a Multi-centre Study. Cardiovasc. Interv. Radiol..

[84] Heppt M.V., Steeb T., Schlager J.G., Rosumeck S., Dressler C., Ruzicka T., Nast A., Berking C. (2017). Immune checkpoint blockade for unresectable or metastatic uveal melanoma: A systematic review. Cancer Treat. Rev..

[85] Klemen N.D., Wang M., Rubinstein J.C., Olino K., Clune J., Ariyan S., Cha C., Weiss S.A., Kluger H.M., Sznol M. (2020). Survival after checkpoint inhibitors for metastatic acral, mucosal and uveal melanoma. J. Immunother. Cancer.

[86] Rozeman E.A., Prevoo W., Meier M.A.J., Sikorska K., Van T.M., van de Wiel B.A., van der Wal J.E., Mallo H.A., Grijpink-Ongering L.G., Broeks A. (2020). Phase Ib/II trial testing combined radiofrequency ablation and ipilimumab in uveal melanoma (SECIRA-UM). Melanoma Res..

[87] Heppt M.V., Amaral T., Kahler K.C., Heinzerling L., Hassel J.C., Meissner M., Kreuzberg N., Loquai C., Reinhardt L., Utikal J. (2019). Combined immune checkpoint blockade for metastatic uveal melanoma: A retrospective, multi-center study. J. Immunother. Cancer.

[88] Karivedu V., Eldessouki I., Taftaf A., Zhu Z., Makramalla A., Karim N.A. (2019). Nivolumab and Ipilimumab in the Treatment of Metastatic Uveal Melanoma: A Single-Center Experience. Case Rep. Oncol. Med..

[89] Rossi E., Pagliara M.M., Orteschi D., Dosa T., Sammarco M.G., Caputo C.G., Petrone G., Rindi G., Zollino M., Blasi M.A. (2019). Pembrolizumab as first-line treatment for metastatic uveal melanoma. Cancer Immunol. Immunother. CII.

[90] Namikawa K., Takahashi A., Mori T., Tsutsumida A., Suzuki S., Motoi N., Jinnai S., Kage Y., Mizuta H., Muto Y. (2020). Nivolumab for patients with metastatic uveal melanoma previously untreated with ipilimumab: A single-institution retrospective study. Melanoma Res..

[91] Arzu Yasar H., Turna H., Esin E., Murat Sedef A., Alkan A., Oksuzoglu B., Ozdemir N., Sendur M.N., Sezer A., Kilickap S. (2020). Prognostic factors for survival in patients with mucosal and ocular melanoma treated with ipilimumab: Turkish Oncology Group study. J. Oncol. Pharm. Pract..

[92] Kirchberger M.C., Moreira A., Erdmann M., Schuler G., Heinzerling L. (2018). Real world experience in low-dose ipilimumab in combination with PD-1 blockade in advanced melanoma patients. Oncotarget.

[93] Heppt M.V., Heinzerling L., Kahler K.C., Forschner A., Kirchberger M.C., Loquai C., Meissner M., Meier F., Terheyden P., Schell B. (2017). Prognostic factors and outcomes in metastatic uveal melanoma treated with programmed cell death-1 or combined PD-1/cytotoxic T-lymphocyte antigen-4 inhibition. Eur. J. Cancer.

[94] Bender C., Enk A., Gutzmer R., Hassel J.C. (2017). Anti-PD-1 antibodies in metastatic uveal melanoma: A treatment option?. Cancer Med..

[95] Okada M., Kijima T., Aoe K., Kato T., Fujimoto N., Nakagawa K., Takeda Y., Hida T., Kanai K., Imamura F. (2019). Clinical Efficacy and Safety of Nivolumab: Results of a Multicenter, Open-label, Single-arm, Japanese Phase II study in Malignant Pleural Mesothelioma (MERIT). Clin. Cancer Res..

[96] Pelster M., Gruschkus S.K., Bassett R., Gombos D.S., Shephard M., Posada L., Glover M., Diab A., Hwu P., Patel S.P. (2019). Phase II study of ipilimumab and nivolumab (ipi/nivo) in metastatic uveal melanoma (UM).in metastatic uveal melanoma (UM). J. Clin. Oncol..

[97] Algazi A.P., Tsai K.K., Shoushtari A.N., Munhoz R.R., Eroglu Z., Piulats J.M., Ott P.A., Johnson D.B., Hwang J., Daud A.I. (2016). Clinical outcomes in metastatic uveal melanoma treated with PD-1 and PD-L1 antibodies. Cancer.

[98] Danielli R., Ridolfi R., Chiarion-Sileni V., Queirolo P., Testori A., Plummer R., Boitano M., Calabro L., Rossi C.D., Giacomo A.M. (2012). Ipilimumab in pretreated patients with metastatic uveal melanoma: Safety and clinical efficacy. Cancer Immunol. Immunother. CII.

[99] Joshua A.M., Monzon J.G., Mihalcioiu C., Hogg D., Smylie M., Cheng T. (2015). A phase 2 study of tremelimumab in patients with advanced uveal melanoma. Melanoma Res..

[100] Karydis I., Chan P.Y., Wheater M., Arriola E., Szlosarek P.W., Ottensmeier C.H. (2016). Clinical activity and safety of Pembrolizumab in Ipilimumab pre-treated patients with uveal melanoma. Oncoimmunology.

[101] Kelderman S., van der Kooij M.K., van den Eertwegh A.J., Soetekouw P.M., Jansen R.L., van den Brom R.R., Hospers G.A., Haanen J.B., Kapiteijn E., Blank C.U. (2013). Ipilimumab in pretreated metastastic uveal melanoma patients. Results of the Dutch Working group on Immunotherapy of Oncology (WIN-O). Acta Oncol..

[102] Luke J.J., Callahan M.K., Postow M.A., Romano E., Ramaiya N., Bluth M., Giobbie-Hurder A., Lawrence D.P., Ibrahim N., Ott P.A. (2013). Clinical activity of ipilimumab for metastatic uveal melanoma: A retrospective review of the Dana-Farber Cancer Institute, Massachusetts General Hospital, Memorial Sloan-Kettering Cancer Center, and University Hospital of Lausanne experience. Cancer.

[103] Maio M., Danielli R., Chiarion-Sileni V., Pigozzo J., Parmiani G., Ridolfi R., De Rosa F., Del Vecchio M., Di Guardo L., Queirolo P. (2013). Efficacy and safety of ipilimumab in patients with pre-treated, uveal melanoma. Ann. Oncol..

[104] van der Kooij M.K., Joosse A., Speetjens F.M., Hospers G.A., Bisschop C., de Groot J.W., Koornstra R., Blank C.U., Kapiteijn E. (2017). Anti-PD1 treatment in metastatic uveal melanoma in the Netherlands. Acta Oncol..

[105] Zimmer L., Vaubel J., Mohr P., Hauschild A., Utikal J., Simon J., Garbe C., Herbst R., Enk A., Kampgen E. (2015). Phase II DeCOG-study of ipilimumab in pretreated and treatment-naive patients with metastatic uveal melanoma. PLoS ONE.

[106] Piulats J.M., Cruz-Merino L.D.L., Garcia M.T.C., Berrocal A., Alonso-Carrión L., Espinosa E., Castro R.L., Rodriguez-Abreu D., Fra P.L., Martin-Algarra S. (2017). Phase II multicenter, single arm, open label study of nivolumab (NIVO) in combination with ipilimumab (IPI) as first line in adult patients (pts) with metastatic uveal melanoma (MUM): GEM1402 NCT02626962. J. Clin. Oncol..

[107] Hodi F.S., O’Day S.J., McDermott D.F., Weber R.W., Sosman J.A., Haanen J.B., Gonzalez R., Robert C., Schadendorf D., Hassel J.C. (2010). Improved survival with ipilimumab in patients with metastatic melanoma. N. Engl. J. Med..

[108] Triozzi P.L., Eng C., Singh A.D. (2008). Targeted therapy for uveal melanoma. Cancer Treat. Rev..

[109] Onken M.D., Worley L.A., Long M.D., Duan S., Council M.L., Bowcock A.M., Harbour J.W. (2008). Oncogenic mutations in GNAQ occur early in uveal melanoma. Investig. Ophthalmol. Vis. Sci..

[110] Van Raamsdonk C.D., Griewank K.G., Crosby M.B., Garrido M.C., Vemula S., Wiesner T., Obenauf A.C., Wackernagel W., Green G., Bouvier N. (2010). Mutations in GNA11 in uveal melanoma. N. Engl. J. Med..

[111] Shah S., Luke J.J., Jacene H.A., Chen T., Giobbie-Hurder A., Ibrahim N., Buchbinder E.L., McDermott D.F., Flaherty K.T., Sullivan R.J. (2018). Results from phase II trial of HSP90 inhibitor, STA-9090 (ganetespib), in metastatic uveal melanoma. Melanoma Res..

[112] Mahipal A., Tijani L., Chan K., Laudadio M., Mastrangelo M.J., Sato T. (2012). A pilot study of sunitinib malate in patients with metastatic uveal melanoma. Melanoma Res..

[113] Bhatia S., Moon J., Margolin K.A., Weber J.S., Lao C.D., Othus M., Aparicio A.M., Ribas A., Sondak V.K. (2012). Phase II trial of sorafenib in combination with carboplatin and paclitaxel in patients with metastatic uveal melanoma: SWOG S0512. PLoS ONE.

[114] Mouriaux F., Servois V., Parienti J.J., Lesimple T., Thyss A., Dutriaux C., Neidhart-Berard E.M., Penel N., Delcambre C., Peyro Saint Paul L. (2016). Sorafenib in metastatic uveal melanoma: Efficacy, toxicity and health-related quality of life in a multicentre phase II study. Br. J. Cancer.

[115] Niederkorn A., Wackernagel W., Artl M., Schwantzer G., Aigner B., Richtig E. (2014). Response of patients with metastatic uveal melanoma to combined treatment with fotemustine and sorafenib. Acta Ophthalmol..

[116] Hofmann U.B., Kauczok-Vetter C.S., Houben R., Becker J.C. (2009). Overexpression of the KIT/SCF in uveal melanoma does not translate into clinical efficacy of imatinib mesylate. Clin. Cancer Res..

[117] Penel N., Delcambre C., Durando X., Clisant S., Hebbar M., Negrier S., Fournier C., Isambert N., Mascarelli F., Mouriaux F. (2008). O-Mel-Inib: A Cancero-pole Nord-Ouest multicenter phase II trial of high-dose imatinib mesylate in metastatic uveal melanoma. Investig. New Drugs.

[118] Daud A., Kluger H.M., Kurzrock R., Schimmoller F., Weitzman A.L., Samuel T.A., Moussa A.H., Gordon M.S., Shapiro G.I. (2017). Phase II randomised discontinuation trial of the MET/VEGF receptor inhibitor cabozantinib in metastatic melanoma. Br. J. Cancer.

[119] Ali M.M., Roe S.M., Vaughan C.K., Meyer P., Panaretou B., Piper P.W., Prodromou C., Pearl L.H. (2006). Crystal structure of an Hsp90-nucleotide-p23/Sba1 closed chaperone complex. Nature.

[120] Stebbins C.E., Russo A.A., Schneider C., Rosen N., Hartl F.U., Pavletich N.P. (1997). Crystal structure of an Hsp90-geldanamycin complex: Targeting of a protein chaperone by an antitumor agent. Cell.

[121] Whitesell L., Lindquist S.L. (2005). HSP90 and the chaperoning of cancer. Nat. Rev. Cancer.

[122] Faingold D., Marshall J.C., Antecka E., Di Cesare S., Odashiro A.N., Bakalian S., Fernandes B.F., Burnier M.N. (2008). Immune expression and inhibition of heat shock protein 90 in uveal melanoma. Clin. Cancer Res..

[123] Jhaveri K., Modi S. (2015). Ganetespib: Research and clinical development. Oncotargets Ther..

[124] Sato T. (2010). Locoregional management of hepatic metastasis from primary uveal melanoma. Semin. Oncol..

[125] Levey A.O., Elsayed M., Lawson D.H., Ermentrout R.M., Kudchadkar R.R., Bercu Z.L., Yushak M.L., Newsome J., Kokabi N. (2020). Predictors of Overall and Progression-Free Survival in Patients with Ocular Melanoma Metastatic to the Liver Undergoing Y90 Radioembolization. Cardiovasc. Interv. Radiol..

[126] Ponti A., Denys A., Digklia A., Schaefer N., Hocquelet A., Knebel J.F., Michielin O., Dromain C., Duran R. (2020). First-Line Selective Internal Radiation Therapy in Patients with Uveal Melanoma Metastatic to the Liver. J. Nucl. Med..

[127] Gonsalves C.F., Eschelman D.J., Adamo R.D., Anne P.R., Orloff M.M., Terai M., Hage A.N., Yi M., Chervoneva I., Sato T. (2019). A Prospective Phase II Trial of Radioembolization for Treatment of Uveal Melanoma Hepatic Metastasis. Radiology.

[128] Zheng J., Irani Z., Lawrence D., Flaherty K., Arellano R.S. (2018). Combined Effects of Yttrium-90 Transarterial Radioembolization around Immunotherapy for Hepatic Metastases from Uveal Melanoma: A Preliminary Retrospective Case Series. J. Vasc. Interv. Radiol. JVIR.

[129] Xing M., Prajapati H.J., Dhanasekaran R., Lawson D.H., Kokabi N., Eaton B.R., Kim H.S. (2017). Selective Internal Yttrium-90 Radioembolization Therapy (90Y-SIRT) Versus Best Supportive Care in Patients With Unresectable Metastatic Melanoma to the Liver Refractory to Systemic Therapy: Safety and Efficacy Cohort Study. Am. J. Clin. Oncol..

[130] Eldredge-Hindy H., Ohri N., Anne P.R., Eschelman D., Gonsalves C., Intenzo C., Bar-Ad V., Dicker A., Doyle L., Li J. (2016). Yttrium-90 Microsphere Brachytherapy for Liver Metastases From Uveal Melanoma: Clinical Outcomes and the Predictive Value of Fluorodeoxyglucose Positron Emission Tomography. Am. J. Clin. Oncol..

[131] Klingenstein A., Haug A.R., Zech C.J., Schaller U.C. (2013). Radioembolization as locoregional therapy of hepatic metastases in uveal melanoma patients. Cardiovasc. Interv. Radiol..

[132] Schelhorn J., Richly H., Ruhlmann M., Lauenstein T.C., Theysohn J.M. (2015). A single-center experience in radioembolization as salvage therapy of hepatic metastases of uveal melanoma. Acta Radiol. Open.

[133] Sato T., Eschelman D.J., Gonsalves C.F., Terai M., Chervoneva I., McCue P.A., Shields J.A., Shields C.L., Yamamoto A., Berd D. (2008). Immunoembolization of malignant liver tumors, including uveal melanoma, using granulocyte-macrophage colony-stimulating factor. J. Clin. Oncol..

[134] Bjornsti M.A., Houghton P.J. (2004). The TOR pathway: A target for cancer therapy. Nat. Rev. Cancer.

[135] Abdel-Rahman M.H., Yang Y., Zhou X.P., Craig E.L., Davidorf F.H., Eng C. (2006). High frequency of submicroscopic hemizygous deletion is a major mechanism of loss of expression of PTEN in uveal melanoma. J. Clin. Oncol..

[136] O’Reilly K.E., Rojo F., She Q.B., Solit D., Mills G.B., Smith D., Lane H., Hofmann F., Hicklin D.J., Ludwig D.L. (2006). mTOR inhibition induces upstream receptor tyrosine kinase signaling and activates Akt. Cancer Res..

[137] Girnita A., All-Ericsson C., Economou M.A., Astrom K., Axelson M., Seregard S., Larsson O., Girnita L. (2006). The insulin-like growth factor-I receptor inhibitor picropodophyllin causes tumor regression and attenuates mechanisms involved in invasion of uveal melanoma cells. Clin. Cancer Res..

[138] All-Ericsson C., Girnita L., Seregard S., Bartolazzi A., Jager M.J., Larsson O. (2002). Insulin-like growth factor-1 receptor in uveal melanoma: A predictor for metastatic disease and a potential therapeutic target. Investig. Ophthalmol. Vis. Sci..

[139] Shoushtari A.N., Ong L.T., Schoder H., Singh-Kandah S., Abbate K.T., Postow M.A., Callahan M.K., Wolchok J., Chapman P.B., Panageas K.S. (2016). A phase 2 trial of everolimus and pasireotide long-acting release in patients with metastatic uveal melanoma. Melanoma Res..

[140] Bale R., Schullian P., Schmuth M., Widmann G., Jaschke W., Weinlich G. (2016). Stereotactic Radiofrequency Ablation for Metastatic Melanoma to the Liver. Cardiovasc. Interv. Radiol..

[141] Eichler K., Zangos S., Gruber-Rouh T., Vogl T.J., Mack M.G. (2014). MR-guided laser-induced thermotherapy (LITT) in patients with liver metastases of uveal melanoma. J. Eur. Acad. Dermatol. Venereol. JEADV.

[142] Bol K.F., van den Bosch T., Schreibelt G., Mensink H.W., Keunen J.E., Kilic E., Japing W.J., Geul K.W., Westdorp H., Boudewijns S. (2016). Adjuvant Dendritic Cell Vaccination in High-Risk Uveal Melanoma. Ophthalmology.

[143] Bol K.F., Mensink H.W., Aarntzen E.H., Schreibelt G., Keunen J.E., Coulie P.G., de Klein A., Punt C.J., Paridaens D., Figdor C.G. (2014). Long overall survival after dendritic cell vaccination in metastatic uveal melanoma patients. Am. J. Ophthalmol..

[144] Uusi-Kerttula H., Hulin-Curtis S., Davies J., Parker A.L. (2015). Oncolytic Adenovirus: Strategies and Insights for Vector Design and Immuno-Oncolytic Applications. Viruses.

[145] Alemany R., Cascallo M. (2009). Oncolytic viruses from the perspective of the immune system. Future Microbiol..

[146] Garcia M., Moreno R., Gil-Martin M., Cascallo M., de Olza M.O., Cuadra C., Piulats J.M., Navarro V., Domenech M., Alemany R. (2019). A Phase 1 Trial of Oncolytic Adenovirus ICOVIR-5 Administered Intravenously to Cutaneous and Uveal Melanoma Patients. Hum. Gene Ther..

[147] Fournier G.A., Albert D.M., Arrigg C.A., Cohen A.M., Lamping K.A., Seddon J.M. (1984). Resection of solitary metastasis. Approach to palliative treatment of hepatic involvement with choroidal melanoma. Arch. Ophthalmol..

[148] Hsueh E.C., Essner R., Foshag L.J., Ye X., Wang H.J., Morton D.L. (2004). Prolonged survival after complete resection of metastases from intraocular melanoma. Cancer.

[149] Frenkel S., Nir I., Hendler K., Lotem M., Eid A., Jurim O., Pe’er J. (2009). Long-term survival of uveal melanoma patients after surgery for liver metastases. Br. J. Ophthalmol..

[150] Aoyama T., Mastrangelo M.J., Berd D., Nathan F.E., Shields C.L., Shields J.A., Rosato E.L., Rosato F.E., Sato T. (2000). Protracted survival after resection of metastatic uveal melanoma. Cancer.

[151] Kodjikian L., Grange J.D., Rivoire M. (2005). Prolonged survival after resection of liver metastases from uveal melanoma and intra-arterial chemotherapy. Graefes Arch. Clin. Exp. Ophthalmol..

[152] Rivoire M., Kodjikian L., Baldo S., Kaemmerlen P., Negrier S., Grange J.D. (2005). Treatment of liver metastases from uveal melanoma. Ann. Surg. Oncol..

[153] Servois V., Bouhadiba T., Dureau S., Da Costa C., Almubarak M.M., Foucher R., Savignoni A., Cassoux N., Pierron G., Mariani P. (2019). Iterative treatment with surgery and radiofrequency ablation of uveal melanoma liver metastasis: Retrospective analysis of a series of very long-term survivors. Eur. J. Surg. Oncol..

[154] Gomez D., Wetherill C., Cheong J., Jones L., Marshall E., Damato B., Coupland S.E., Ghaneh P., Poston G.J., Malik H.Z. (2014). The Liverpool uveal melanoma liver metastases pathway: Outcome following liver resection. J. Surg. Oncol..

[155] Mariani P., Almubarak M.M., Kollen M., Wagner M., Plancher C., Audollent R., Piperno-Neumann S., Cassoux N., Servois V. (2016). Radiofrequency ablation and surgical resection of liver metastases from uveal melanoma. Eur. J. Surg. Oncol..

[156] Yang X.Y., Xie F., Tao R., Li A.J., Wu M.C. (2013). Treatment of liver metastases from uveal melanoma: A retrospective single-center analysis. Hepatobiliary Pancreat. Dis. Int. HBPD INT.

[157] Mariani P., Piperno-Neumann S., Servois V., Berry M.G., Dorval T., Plancher C., Couturier J., Levy-Gabriel C., Lumbroso-Le Rouic L., Desjardins L. (2009). Surgical management of liver metastases from uveal melanoma: 16 years’ experience at the Institut Curie. Eur. J. Surg. Oncol..

[158] Kivela T., Eskelin S., Kujala E. (2006). Metastatic uveal melanoma. Int. Ophthalmol. Clin..

[159] Kivela T.T., Piperno-Neumann S., Desjardins L., Schmittel A., Bechrakis N., Midena E., Leyvraz S., Zografos L., Grange J.D., Ract-Madoux G. (2016). Validation of a Prognostic Staging for Metastatic Uveal Melanoma: A Collaborative Study of the European Ophthalmic Oncology Group. Am. J. Ophthalmol..

[160] Rietschel P., Panageas K.S., Hanlon C., Patel A., Abramson D.H., Chapman P.B. (2005). Variates of survival in metastatic uveal melanoma. J. Clin. Oncol..

[161] Rantala E.S., Hernberg M., Kivela T.T. (2019). Overall survival after treatment for metastatic uveal melanoma: A systematic review and meta-analysis. Melanoma Res..

[162] Onken M.D., Worley L.A., Ehlers J.P., Harbour J.W. (2004). Gene expression profiling in uveal melanoma reveals two molecular classes and predicts metastatic death. Cancer Res..

[163] Harbour J.W. (2014). A prognostic test to predict the risk of metastasis in uveal melanoma based on a 15-gene expression profile. Methods Mol. Biol..

[164] Jager M.J., Shields C.L., Cebulla C.M., Abdel-Rahman M.H., Grossniklaus H.E., Stern M.H., Carvajal R.D., Belfort R.N., Jia R., Shields J.A. (2020). Uveal melanoma. Nat. Rev. Dis. Primers.

[165] Ewens K.G., Kanetsky P.A., Richards-Yutz J., Purrazzella J., Shields C.L., Ganguly T., Ganguly A. (2014). Chromosome 3 status combined with BAP1 and EIF1AX mutation profiles are associated with metastasis in uveal melanoma. Investig. Ophthalmol. Vis. Sci..

[166] van de Nes J.A., Nelles J., Kreis S., Metz C.H., Hager T., Lohmann D.R., Zeschnigk M. (2016). Comparing the Prognostic Value of BAP1 Mutation Pattern, Chromosome 3 Status, and BAP1 Immunohistochemistry in Uveal Melanoma. Am. J. Surg. Pathol..

[167] Abdel-Rahman M.H., Cebulla C.M., Verma V., Christopher B.N., Carson W.E., Olencki T., Davidorf F.H. (2012). Monosomy 3 status of uveal melanoma metastases is associated with rapidly progressive tumors and short survival. Exp. Eye Res..

[168] Therasse P., Arbuck S.G., Eisenhauer E.A., Wanders J., Kaplan R.S., Rubinstein L., Verweij J., Van Glabbeke M., van Oosterom A.T., Christian M.C. (2000). New guidelines to evaluate the response to treatment in solid tumors. European Organization for Research and Treatment of Cancer, National Cancer Institute of the United States, National Cancer Institute of Canada. J. Natl. Cancer Inst..

[169] Moher D., Liberati A., Tetzlaff J., Altman D.G., Group P. (2009). Preferred reporting items for systematic reviews and meta-analyses: The PRISMA statement. PLoS Med..

[170] AHRQ Agency for Healthcare Research and Quality Website. Agency for Healthcare Research and Quality. https://www.ahrq.gov/research/publications/pubcomguide/index.html.

