# Supplementary material for: Treatment of Metastatic Uveal Melanoma: Systematic Review
Source: Cancers (Basel). 2020 Sep 8;12(9):2557. doi: 10.3390/cancers12092557 (PMC7565536; doi:10.3390/cancers12092557)
Supplement: Supplementary file 1 [file cancers-12-02557-s001.pdf]

cancers

MDPI

Review

Treatment of Metastatic Uveal Melanoma: Systematic Review

Cristina Rodriguez-Vidal, Daniel Fernandez, Beatriz Fernandez-Marta, Nerea Lago, María Pardo, Paula Silva, Laura Paniagua, María José Blanco-Teijeiro, Antonio Piñeiro and Manuel Bande

Supplementary Data

Table S1. Included studies of Conventional Chemotherapy.

Drug / Intervention	Author	Yearofpublication	Studio design	Numberofpatients	Number of first line treatments (%)	Number of patients with surgical	Median from metastases to treatment in months	DefinitionofOS	Median OS published in months (range)	CIOS	AnnualOS	Excepcional outcomes	Definition Free Interval of Progression	Median FIP	CI FIP	Adverse effects
**Multiple***	Pons [20]	2011	Case Study Series Comparative (III)	25	0 (0%)	0 (0%)	NP	NP	10.8	CI 95% (5.4 - 16.3)	NP	__	NP	NP	NP	NP
**Temozolomide**	F. Bol [21]	2019	Case Study Series (III)	32	32 (100%)	NP	NP	TX	5.7	NP	1 year: 18.8%	__	TX	2,5	NP	NP
**Multiple systemic TX not including CPIs****	T. Xu [22]	2019	Case Study Series (III)	14	14 (100%)	NP	NP	TX	10.3	NP	NP	__	TX	4	NP	NP
**Temozolomideor Dacarbazine**	Luke [23]	2019	Phase II Prospective Randomized Trial (Ib)	15	9 (60%)	NP	NP	TX	7.3	CI 95% (5.6 -NP)	NP	__	TX	1.94	CI 95% (1.8 - 5.3)	• AE 15 (100%): Fatigue 7(46.7%), Nausea 7(46.7%), Vomiting 5(33.3%), Platelet fall 4(26.7%)<br>• Grade 3 or 4: 3 (20%): Platelet Drop 2 (13.4%), Diarrhea 1 (6.7%), Nausea 1 (6.7%)

**Chemotherapy*** **	Tulokas [24]	2018	Case Study Series Comparative (III)	8	8 (100%)	0 (0%)	NP	TX	10, 5 (range, 3 - 16.5)	CI 95% (6.8 - 13.3)	1 year: 50%<br>2 years: 0%	—	NP	NP	NP	• AE: nausea, infections, declining performance status, liver and hematological toxicity.
**Cisplatin + Dacarbazine + Vinblastine**	Schinzari [25]	2017	Phase II Prospective Non-Randomised Trial (IIa)	25	25 (100%)	0 (0%)	NP	TX	13	NP	NP	1 patient still alive after first line therapy at 72 months	TX	5.5	NP	• Grades 1 and 2: 9(36%): Nausea and Vomiting 8(32%), Neurotoxicity 8(32%), Anemia 6(24%), Asthenia 6(24%)<br>• Grades 3 and 4: 5(20%): Neutropenia 4(16%), Nausea and Vomiting 2(8%) Anemia 2(8%), Thrombocytopenia 1(4%)
**Dacarbazine**	Carling [26]	2015	Case Study Series Comparative (III)	14	NP	NP	2.5 (range, 0.6-4.7)	TX	4,6	CI 95% (1.4-13.7)	1 year: 25%<br>2 years: 0.05%	—	NP	NP	NP	NP
**Gemcitabine + treosulfan**	Corrie [27]	2005	Phase I Prospective Non-Randomized Test (IIa)	5	4 (80%)	0 (0%)	NP	NP	12,2 (range, 5 – 23.7)	CI 95% (4.6-ND)	1 year: 75%<br>2 years: 0%	—	NP	6.21 (range, 1.61 - 8.74)	ND	NP
**Docosahexaenoicacid-paclitaxel**	Homsi [28]	2010	Phase II Non-Randomized Open Prospective Trial (IIa)	22	13 (59%)	NP	NP	EP	9,8	NP	NP	—	R-P	3 (range, 3-7)	NP	• Pain 16 (73%), Fatigue 14 (64%), Headache 14 (64%), Myalgia 14 (64%)<br>• Severe 3 or 4: Neutropenia 5 (23%), Musculoskeletal pain 2 (10%)
**Fotemustine**	Leyvraz [29]	2014	Prospective multcenter randomized trial (Ib)	85	85 (100%)	0 (0%)	NP	EP	13,8	CI 95% (10.2 - 17.2)	NP	1 patient still alive at 60 months	EP	3,7	CI 95% (2.0–4.1)	• Nausea and vomiting 37 (43.53%) Abdominal pain 33(38, 82%)<br>• Grade 3 or 4: Thrombocytopenia (42.1 %) Neutropenia (62.6 %)
**Gemcitabine + treosulfan**	Pföhler [30]	2003	Retrospective case series (III)	14	1 (7%)	0 (0%)	NP	EP	14 (range, 3-33.2)	CI 95% (12 - 31)	1 year: 80% (95% CI 54-100)	—	EP	6,56 (range, 8-21.5)	CI 95% (2.9-14.2)	• Neutropenia 3 (21%), Thrombocytopenia 9 (64.1%)<br>• Grade 3 or 4: Neutropenia 2 (14%)

																Thrombocytopenia 9 (64.1%)
**Temozolomide + bevacizumab**	Piperno-Neumann [31]	2016	Phase II Prospective Trial (IIb)	35	35 (100%)	0 (0%)	NP	TX	10	CI 95% (8 - 15)	NP	—	NP	3	NP	• Grade 1 or 2 nausea, Constipation, Abdominal pain<br>• Grade 3 or 4: 16 (45.71%): Neutropenia 7 (20%), Rhombocytopenia or Constipation or itchiness 7 (20%), Venous thromboembolism 1 (2.85%), Febrile neutropenia and pneumonitis grade 4 1 (2.85%)
**Gemcitabine + treosulfan + Cisplatin**	Schmittel [32]	2005	Phase II Prospective Non-Randomised Trial (IIa)	19	19 (100%)	NP	NP	TX	7,7	CI 95% (1.9 - 13.8)	1 year: 31%.	—	EP	3	CI 95%, (1.8-3. 1)	• Grade 3 or 4: Leukopenia 9 (47.36%) Thrombocytopenia 8 (42.1%) Anemia 3 (15.79%)
**Gemcitabine + treosulfan 2500 or 3000 mg/m2**	Schmittel [33]	2005	Phase II Prospective Non-Randomised Trial (IIa)	14	12 (86%)	NP	NP	TX	6	95% CI (4 - 8)	1 year: 7.1	—	EP	2	CI 95%, (0.6-3.4)	• Grade 3 or 4: Leukopenia 1 (14%) Thrombocytopenia 5 (36%), Anemia 0 (0%)
**Gemcitabine + treosulfan 3500 or 4000 mg/m2**				19	16 (84%)	NP	NP		9	CI 95% (0 - 18)	1 year: 47.3%			3	95% CI (1.7–4.3)	• Grade 3 or 4: Leukopenia 4 (21%), Thrombocytopenia 5 (26%), Anemia 1 (5%)
**Gemcitabine + treosulfan**	Terheyden [34]	2004	Phase II Prospective Non-Randomised Trial (IIa)	20	8 (40%)	0 (0%)	NP	TX	17 (range, 1 - 57)	CI 95% (8.8-25.2)	1 year: 65% 2 years: 35% 3 years: 20%	1 patient still alive at 57 months	TX	6.16 (range, 6-10.4)	NP	• Grade 3 or 4: Leukopenia 1 (20%), Thrombocytopenia 1 (20%), Anemia 1 (20%)

AE, adverse effect; CI, confidence interval; CPI, checkpoint inhibitors;EP, enrollment period; ND, not defined; NP, not provided; R-P, response-progression; OS, overall survival;TX, treatment， * Thirteen patients received dacarbazine, five received temozolomide with or without interferon, five received fotemustine, two received carboplatin/dacarbazine/interferon alpha/interleukin-2. Due to multiple different therapies, patients were included in the conventional chemotherapy

group, but could not be arranged under a specific chemotherapy agent. **Carboplatin with paclitaxel, dacarbazine, temozolomide, sunitinib and interferon with stibogluconate， *** 7 patients received dacarbazine as 1st line and the other patient received docetaxel + cisplatin.

Table S2. Included studies of Chemoimmunotherapy.

rug / Intervention	Author	Yearofpublication	Studio design	Numberofpatients	Number of first line treatments (%)	Number of patients with surgical treatment (%)	Median from metastases to treatment in months	DefinitionofOS	Median OS published in months (range)	CIOS	AnnualOS	Excepcional Outcomes	Definition Free Interval of Progression	Median FIP	CI FIP	Adverse effects
**Selumetenib + Dacarbazine**	Carvajal [39]	2018	Phase II Prospective Randomized Trial (Ib)	97	97 (100%)	0 (0%)	NP	EP	6.5 approx.	NP	NP	—	EP	2.8	NP	• AE 97 (100%): Nausea 60(62%), Rash 55(57%), Fatigue 43(44%), Diarrhea 43 (44%).<br>• Grade 3 or 4: 60 (61%): Neutropenia 19 (19%), Thrombocytopenia 12(12%), ETS 8(8%), LFT alteration 6(6%).
**Placebo + Dacarbazine**				32	32 (100%)	0 (0%)			5.9 approx.					1.8		
**Fotemustine IA/IV + IL2 + IFN-alpha2**	Becker [40]	2002	Prospective non-randomized comparative trial (IIa)	48	NP	0 (0%)	NP	TX	12	95% CI (11,4 - 12,8)	NP	—	TX	12 (range 5.1-28.1)	NP	• Photomustine IA: Leucopenia 2 (4.17%), Thrombocytopenia 4 (8.33%), ComplCIations derived from the intra-arterial catheter:<br>-Erosive gastritis 1 (2.08%)<br>-GastrCI Ulcer 1 (2.08%)<br>-Arterial wall dissection 1 (2.08%)<br>-Angiospasm 1 (2.08%)<br>• Photomustine IV: Leucopenia 6 (12.5%), Thrombocytopenia 12 (25%)<br>• Immunotherapy Grade 3 or 4: Cold 8 (16.7%), Fatigue 6 (12.5%), Hypotension 5 (10.41%)
**BOLD + IFN-alpha2b**	Kivelä[41]	2003	Phase II Non-Randomized Prospective Multicenter Trial (IIa)	24	24 (100%)	0 (0%)	NP	TX	10,6	CI 95% (6.9-16.4)	NP	—	TX	1.9	CI 95% (1.8-3.4)	• Grades 1 and 2: Nausea 15 (63%), Fever 18(75%), Flu-like syndrome 21 (87%), Alopecia 4(46%), Liver Toxicity 19 (80%)<br>• Grade 3 or 4: Alopecia 12 (51%), Neurotoxicity 12 (51%)

**BOLD + leucocyte IFN**	Pyrhönen[42]	2002	Phase II Prospective non-Randomised Trial (IIa)	22	18 (82%)	0 (0%)	NP	TX	12	CI 95% (8-22)	NP	—	TX	4	95% CI (2-10)	• Alopecia (90%), Leukopenia (95%), Liver Toxicity (50%)<br>• Grade 3 or 4: Leukopenia (40%), Alopecia (25%), Thrombocytopenia (20%), Constipation (15%)<br>• Deaths: Myocardial Infarction 1 (4.55%), Sepsis 1 (4.55%)
**Thalidomide + IFN-alpha2b**	Solti[43]	2007	Prospective non-randomised comparative pilot study (IIa)	6	0 (0%)	NP	NP	TX	3,7 (range, 0.9-ND)	CI 95% (0.9 -ND)	1 year: 16.6% 2 years: 16.6%	—	TX	1.7 (range, 0.8-14)	95% CI (1.3-2)	Not specified for uveal melanoma
**Fotemustine IV + IL2 + IFN-alpha2b**	Terheyden[44]	1998	Case Study Series (III)	3	3 (100%)	0 (0%)	2 (range, 2-4)	TX	ND	ND	1 year: 100% 2 years: 66.7% 3 years 66.7%:	1 patient alive at 57 months	TX	8 (range, 8-49)	ND	NP
**Dacarbazine + IFN-alpha2a + bevacizumab**	Vihinen[45]	2010	Phase II Prospective Trial (IIb)	4	4 (100%)	0 (0%)	NP	TX	10.8 (range ,6.5-ND)	CI 95% (6.5–ND)	1 year: 25% 2 years: 25% 3 years 25%:	—	TX	1.6 (range, 1.6-8.1)	ND	• Grades 1 and 2: Diarrhoea 2 (50%), Hoarseness (50%), Fatigue 2 (50%)<br>• Grade 3 or 4: Hypertension intracerebralhaemorrhaged

AE, adverse effect; CI, confidence interval; CPI, checkpoint inhibitors; EP, enrollment period; IA, intra-arterial; IV, intravenous;ND, not defined; NP, not provided; OS, overall survival; TX, treatment.

Table S3. Included studies of Intra-arterial Liver Chemotherapy (ILC).

Drug / Intervention	Author	Yearofpublication	Studio design	Numberofpatients	Number of first line treatments (%)	Number of patients with surgical [illegible]	Median from metastases to treatment in months	DefinitionofOS	Median OS published in months (range)	CIOS	AnnualOS	Exceptional outcome	Definition Free Interval of Progression	Median FIP	CI FIP	Adverse effects
**Fotemustine**	Leyvraz [29]	2014	Prospective multicenter randomized trial (Ib)	86	86 (100%)	0(0%)	NP	EP	14,6	CI 95% (10.2-15.4)	NP	1 patient still alive at 60 months	EP	4.5	CI95% (4.1–6.0)	(28 patients evaluated)

																• Grade >3: Neutropenia 6 (21%), Thrombocytopenia 6(21%),<br>Transient asymptomatic elevation of glutamyltransferaseγ(>x5 normal levels): 13 (48%)
**Melphalan**	Boone [49]	2018	Case Study Series (III)	14	10 (71,42%)	NP	NP	TX	2,9 (range, 0.2-15.8)	CI 95% (0.2 – 5.6)	1 year: 7.1%<br>2 years: 0%	—	NP	NP	NP	NP
**Fotemustine + IPC**	Itchins [50]	2017	Case Study Series (III)	12	NP	NP	NP	DX	19	CI 95% (13.3 - NP)	NP	—	NP	NP	NP	(No detailed information)
**Carboplatin**	Cantore [51]	1994	Phase II Prospective Non-Randomised Trial (IIa)	8	5 (63%)	NP	NP	TX	15* (range, 8-29).	CI 95% (7.0-20.0) *	1 year: 62.5%<br>2 years: 25%	—	NP	NP	NP	• Myelosuppression (most frequent), Nausea and vomiting, Mild epigastric pain 2 (25%), Gastric ulcer 1 (12.5%), Altered liver function tests 3 (37.5%)
**Fotemustine**	Egerer [52]	2001	Non-Randomized Prospective Pilot Study(IIa)	7	5 (71%)	0(0%)	2 (range, 1-10)	TX	18**	CI 95% (14.4 – 25.6) **	1 year: 71.42%<br>2 years: 28.6%<br>3 years: 14.3%	—	TX	16 (range, 0-43)	NP	• Anemia 7(100%), Nausea and Vomiting 7(100%) Thrombocytopenia 7(100%)<br>• Grade >3:Thrombocytopenia 1(14.28%)
**Fotemustineorcarboplatin**	Farolfi [53]	2011	Case Study Series (III)	18	NP	NP	NP	TX	21	CI 95% (8 - 39)	NP	—	TX	6.2	CI 95% (3.7-10.5)	• 15 (65.2%) some form of adverse event:10 (43.5%) grade 1/2 Toxicity, 5 (21.7%) grade 3 hematological toxicity (carboplatin).
**Melphalan**<br>**Melphalan and additional agents*** **	Heusner [54]	2011	Case Study Series Comparative (III)	38<br>23	43 (70%)	NP<br>NP	3.3 (range, 0.5-2)	TX	10.3<br>8.7	CI 95% (5.0-15.6)<br>CI 95% (8.1-9.3)	NP	—	NP	NP	NP	• Thrombocytopenia 30 (49.2%), Leucopenia 21 (34.4%), Neutropenia 1 (1.6%)<br>• Death from liver failure after treatment 1 (1.6%)
**Fotemustine**	Leyvraz [55]	1997	Prospective non-randomized trial (IIa)	31	31 (100%)	NP	NP	DX	14	NP	1 year: 60%<br>2 years: 22.6%	—	11	NP	NP	• Grade 1 and 2: Nausea and Vomiting, Abdominal Pain<br>• Grade 1 and 4: Thrombocytopenia,

																Neutropenia, Elevation of y-glutamyltransferase
**Cisplatin + Vinblastine + Dacarbazine**	Melichar [56]	2009	Case Study Series (III)	10	7 (70%)	0(0%)	1.4 (range, 0.5-9.5)	TX	16	CI 95% (5.0-19.0)	1 year: 60%<br>2 years: 20%<br>3 years: 10 %	1 patient alive until 69 months	NP	NP	NP	NP
**Fotemustine or Dacarbazine + Cisplatin, post-biopsia**[†]	Salmon [57]	1998	Prospective non-randomized comparative case series (III)	16	16(100%)	0(0%)	NP	NP	NP	NP	NP	—	NP	NP	NP	NP
**Fotemustine**	Siegel [58]	2007	Case Study Series (III)	18	NP	0(0%)	22 (range, 6-63)	TX	22	NP	NP	—	NP	NP	NP	• Grade 1 and 2: Nausea and Vomiting (17%), Abdominal Pain (7%)<br>• Grade>3: Thrombocytopenia (30%), Neutropenia (7%)
**Fotemustine**[††]	Peters[59]	2006	Case Study Series (III)	101	101 (100%) NC	39 (39%) ‡	1.9 (range, 0.1-45)	DX	15	CI 95% (12.1-17.6)	1 year: 67% (95% CI 57-75%).<br>2 years: 29%.<br>3 years: 12%	—	NP	NP	NP	• Organic damage: 2 (2%), Pancretaitis 1 (1%), Sclerosing cholangitis 1 (1%)<br>• Grade >3 (11%):,Hematotoxicity (4%)<br>• Risks associated with early intraarterial catheter 9 (9%): Thrombosis 2 (2%), Stenosis / Obstruction 2 (2%), Dislocation 2 (2%)<br>• Risks associated with intra-arterial catheter after several months (catheter thrombosis) 21 (21%)<br>• Post-surgical jaundice 1 (1%)

Approx, approximate; AE, adverse effect; CI, confidence interval; CPI, checkpoint inhibitors; DX, diagnosis; NC, non-chemo, no prior systemic treatment; NP, not provided; LFT, liver function tests; EP, enrollment period; R-P, response-progression; OS, overall survival; TX, treatment. * The median survival is 12 months when calculated from Table 1; the median proportionate survival of 15 months appears to correspond to the median survival. The CI OS: CI 95% (7.0-20.0) is calculated

from Table 1 too. ** The median 18 seems to be calculated arithmetically and not as a Kaplan-Meier estimate.The median survival is 20 months when calculated from Table 1; The CI OS :CI 95% (14.4-25.6) is calculated from Table 1 too. *** Dacarbazine, doxorubicin, fotemustine, gemcitabine, mitomycin C. † Patients received at least two months of fotemustine or dacarbazine and cisplatin after the biopsy. † † In addition, 38 patients underwent volume reduction surgery at the time of catheter placement. In the Kaplan-Meier diagram, 100 patients were represented. ‡ Previous treatment of 11 patients is unknown.

Table S4. Included studies of Transarterial Liver Chemoembolization (TACE).

Drug / Intervention	Author	Yearofpublication	Studio design	Numberofpatients	Number of first line treatments (%)	Number of patients with surgical treatment (%)	Median from metastases to treatment in months	DefinitionofOS	Median OS published in months (range)	CIOS	AnnualOS	Exceptional Outcomes	Definition Free Interval of Progression	Median FIP	CI FIP	Adverse effects
Irinotecan-elutingbeads	Carling [26]	2015	Comparative retrospective case series (III)	14	11 (79%)	1 (7%)	4.1 (range, 1.2-12.7)	TX	9.4 (range, 3-39)	NP	1 year: 25%. 2 years: 20%.	—	NP	NP	NP	• 4 patients: total liver volume increasing between 62.5 and 31.1 % post treatment<br>• 1 patient died 10 days after the fourth procedure, due to disseminated intravascular coagulation (DIC) and multiple cerebral infarctions.
BCNU + gelatinsponge	Patel [60]	2005	Phase II Prospective Trial (IIb)	30	24 (80%)	0 (0%)	NP	TX	5,2 (range, 0,1-27,6)	NP	NP	—	TX	6.7 (range, 2.5-20.2)	NP	• Grade 1 and 2: Liver pain, hiccups, nausea and/or vomiting 7 (23%)<br>• Grade 3 or 4: 8 (26.66%): Hepatic vein thrombosis 2 (6.66%), portal vein thrombosis 1 (3.33%), splenic infarction 1 (3.33%), thrombocytopenia 1 (3.33%), acute renal failure 1 (3.33%), liver failure 3 (10%)

Mitomycin C	Farshid [61]	2013	Case Study Series (III)	20	NP	NP	NP	TX	20	NP	1 year: 73%. 2 years: 56%. 5 years: 28%	—	NP	21 approx	NP	NP
Cisplatinwith/withoutpolyvinylsponge	Agarwala [62]	2004	Phase I/II Prospective Randomized Trial (Ib)	19	13 (68%)	0 (0%)	NP	NP	8,5	NP	NP	—	NP	NP	NP	NP
Cisplatin + Doxorubicin + MMC + gelatinsponge	Dayani [63]	2009	Case Study Series (III)	21	17 (81%)	1 (5%)	NP	TX	7.6‡	NP	NP	—	NP	NP	NP	NP
Fotemustine + polyvinyl alcohol particles	Edelhauser[64]	2012	Case Study Series (III)	21	2 (10%)	0 (0%)	NP	DX	28.7	CI 95% (4.2-53)	NP	—	TX	7.3	CI 95%, (3.3-11.3)	• Fever 4 (19.05%), Pain 3 (14%), Nausea 5 (23.81%)
Irinotecan-elutingbeads	Fiorentini [65]	2009	Phase II Prospective Trial (IIb)	10	0 (0%)	0 (0%)	44 (range, 24-84)	TX	NR‡‡	NP	NP	------	NP	NP	NP	• Grade 2 abdominal pain (NP%)<br>• Grade 3 abdominal pain (NP%), Transient paralytic ileus 1 (10%) Hepatitis without jaundice 4 (40%)
BCNU + gelatinsponge	Gonsalves [66]	2015	Case Study Series (III)	50**	50 (100%)	0 (0%)	NP	TX	7,1 (range, 1,2-32,3)	NP	1 year: 22%	—	TX	5 (range, 1.1-32.3).	NP	• Pain, weight loss or fatigue: 35 (70%)<br>• Asymptomatic transaminitis grade 4: 13 (26%)<br>• No treatment related deaths or major complications.
Cisplatin*** + gelatin sponge or polyvinyl alcohol particles	Gupta [67]	2010	Case Study Series (III)	125	82 (66%)	NP	7(range, 1-122)	TX	6,7	CI 95% (4.9-8.5)	NP	—	TX	3.81	CI 95% (3.15-4.93)	• Anemia and/or thrombocytopenia 16 (12.8%), Ascites with/without anasarca 10 (8%), Bacteremia 7 (5.6%) Tumour lysis syndrome associated

																with renal failure 3 (2.4%)<br>• Deaths 5 (4%): Acute liver and kidney failure 2 (1.6%), Sepsis and airway failure 2 (1.6%), Myocardial infarction 1 (0.8%)
Cisplatin or carboplatin + polyvinyl alcohol particles	Huppert [68]	2010	Prospective non-randomised comparative pilot study (IIa)	14	8 (57%)	1 (7%)	4.5 (range, 1 - 38)	TX	11,5 (range, 3-69)	CI 95% (10,3-13,8)	1 year: 50% 1.5 years: 28 % 2 years: 14%.	1 patient still alive at 69 months	R-P	8.5 (range 5-35)	NP	• Abdominal pain, fever, nausea and vomiting. 14 (100%),<br>• Acute kidney failure 1 (7.14%)
Cisplatin + polyvinylsponge	Mavligit [69]	1988	Case Study Series (III)	30	19 (63%)	0 (0%)	NP	DX	11	95% CI (9-18)	NP	1 patient still alive at 54 months	NP	NP	NP	• Severe abdominal pain associated with transient hepatitis with altered LFT 30 (100%), Transient Hyperbilirubinemia (25%), Transient Paralytic Ileus 1, Significant losses of K and Mg (35%)
Photemistine or cisplatin + starch microspheres	Schuster [70]	2010	Case Study Series (III)	25	0 (0%)	NP	9 (range, 2-24)	TX	6 (range, 0-32)	95% CI (5-7)	1 year: 15%	___	TX	3 (range, 0-9)	95% CI (2-4)	• Grade 1 and 2: 19 (76%): Liver pain 15 (60%), Nausea and/or vomiting 16 (64%), Fatigue 4 (16%), Fever 2 (8%)<br>• Grade 3 or 4: 6: Fever in the absence of outbreak 3 (12%), Splenic infarction 1 (4%), Thrombocytopenia 1 (4%), Gastric ulcer 1 (4%)
Cisplatin + geatinesponge	Shibayama [71]	2017	Case Study Series (III)	29	27 (93%)	1 (3%)	NP	TX	23 (range, 2-47)	NP	1 year: 72.4%. 2 years: 39.4% 5 years: 0%	___	TX	6	NP	• Most common AEs: LFT 29 alloy (100%), Nausea 21 (72.4%), Abdominal pain 18(65.5%) Vomiting 16 (55.2%), Post-embolization syndrome 10(34.5%), Pyrexia 7 (24.1%).

																• Grade 3 or 4 : ALT 15 Elevation (51.7%), AST 10 Elevation (34.5%), Creatinine 1 Elevation (3.4%)
CPT-11 charged microbeads	Valpione [72]	2015	Comparative retrospective case series (III)	58‡‡‡	58 (100%)	0 (0%)	NP	TX	15,5	NP	1 year: 68% 2 years: 34.6	—	NP	NP	NP	• Nausea and/or vomiting 58 (100%), Abdominal pain 43 (74.14%) • Grade 3 or 4: Thrombocytopenia 5 (8.62%), Neutropenia 1 (1.72%)
Blandembolisation + gelatinsponge	Valsecchi [73]	2015	Phase II Randomized Prospective Study (Ib)	27	NP	NP	NP	TX	17,2	CI 95% (11.9-22.4)	NP	1 patient still alive at 60 months	TX	7,1	CI 95% (5-9,1)	• First week: Abdominal pain (38.9%) LFT alteration (17.3%),Haematological (15.3%) -Grade 3 or 4: LFT alteration (1.1%) • From the second week onwards: Abdominal pain (26.9%), LFT alteration (9.6%), haematological (7.4%) -Grade 3or 4: Less than 1%
MMC + iodisedoil + microspheres	Vogl [74]	2007	Prospective non-randomised comparative pilot study (IIa)	12	0 (0%)	4 (33%)	NP	TX	21(range, 5,5-30)	CI 95% (13,3-28,7)	1 year: 75% 2 years: 25%	—	NR	NR	NR	• Mild nausea without vomiting 1 (8.33%) Transient headache 2(16.66%) Transient Fever 3 (25%) • Grade 3 or 4: 0 (0%)

Approx, approximate AE, adverse effect; BCNU,bis-chloroeilnitrosourea; CI, confidence interval; CPI, checkpoint inhibitors; MMC, Mitomycin C; NP, not provided; NR, not reached; LFT, liver function tests; EP, enrollment period; R-P, response-progression; OS, overall survival; TX, treatment. * Out of 19 patients, ten were treated with TACE. ** Fifty patients met the inclusion criteria, but 49 patients are included in the Kaplan-Meier analysis. *** Two patients received additional paclitaxel and one patient doxorubicin + MMC. ‡ Mean survival is calculated instead of median. ‡ ‡ Median overall survival was not reached, i.e. cumulative survival plot did not fall below 50%. ‡ ‡ ‡ Moreover, 49 patients received intravenous fotemustine three weeks after TACE.

Table S5. Included Liver Perfusion Isolation Studies (IHP).

Drug / Intervention	Author	Yearofpublication	Studio design	Numberofpatients	Number of first line treatments (%)	Number of patients with surgical treatment (%)	Median from metastases to treatment in months (range)	DefinitionofOS	Median OS published in months (range)	CIOS	AnnualOS	Exceptional Otcomes	Definition Free Interval of Progression (FIP)	Median FIP	CI FIP	Adverse effects
**Melphalan (percutaneous)**	Artzner [76]	2019	Retrospective case series (III)	16	9 (56.25%)	1 (6.2%)	NP	TX	27,4	CI 95% (4.1-35.4)	1 year: 58%	—	TX	11,1	CI 95% (4.9-23.6)	• Leucopenia 15 (96%), Anemia 15 (96%), Thrombocytopenia 12 (75%), Nausea and Vomiting 10(61%)<br>• Grade 3 or 4: Anemia 4(14%), Leukopenia 4(14%), Thrombocytopenia 4(14%), Cardiovascular Event 1 (4%)
**Melphalan (percutaneous)**	Karydis[77]	2017	Retrospective case series (III)	51	NP	9 (17%)	4.56 (range 1 - 26.3)	TX	15,3	NP	1 year: 64.6%.	—	TX	8,1	NP	• Any AE 51 (100%): Anemia 51(100%), Thrombocytopenia 50(98%), Neutropenia 22(43%), Fatigue 17(33%)<br>• Grades 3 or 4:19(37.5%): Neutropenia 16(31.3%), Thrombocytopenia 16(31.3%), Anemia 15 (29.4%), Cardiovascular events 9(17.6%)
**Melphalan**	Alexander [78]	2003	Phase I/II Prospective Trial (IIb)	29	22 (76%)	0 (0%)	NP	EP	12,1 (range, 3-39)	NP	NP	—	EP	8	NP	• Grade 3 or 4: Transient liver Toxicity 19 (65%)<br>• No treatment related deaths
**Melphalan with/without TNF-alpha or cisplatin**	Ben-Shabat[79]	2016	Retrospective case series (III)	61	Most	NP	NP	TX	22,4	NP	NP	—	NP	NP	NP	• Grade 3 or 4: 6 (9.5%): Respiratory failure 2 (3.2%), Perforated duodenal ulcer (diabetic patient) 1 (1.64%), Transient liver failure 1 (1.64%), Post-operative bleeding 1 (1.64%), Transient liver, respiratory and kidney failure 1 (1.64%)

																• Deaths from liver failure: 5 (8.20%)
**Melphalan**	de Leede[80]	2016	Retrospective case series (III)	31	27 (87%)	0 (0%)	2.3 (range, 0.9-13.3)	TX	10 (range, 0-50)	NP	1 year: 41.9% 2 years: 19.4%. 3 years: 3.5%	1 patient alive at 50 months	TX	6 (range 1-16)	NP	• Veno-occlusive disease 2 (6.45%), Non-infectious fever 1 (3.23%), Liver enzyme elevation, Leucopenia<br>• Deaths from liver failure due to hepatic artery occlusion: 2 (6.45%)
**Melphalan**	Forster [81]	2014	Retrospective case series (III)	5	4 (80%)	0 (0%)	NP	TX	14.2 (range, 10-ND)	CI 95% (10-ND)	1 year: 60% 2 years: 40%. 3 years: 20%	1 patient alive until 46.4 months	TX	7,5(range0-14.9)	CI 95% 13.6 (2.3-24.9)	• Myelosuppression (most common AE), Mild troponin elevations<br>• No treatment related mortality
**Oxaliplatin+melfalan**	van Iersel[82]	2014	Phase I Prospective Trial (IIb)	3	2 (67%)	0 (0%)	NP	TX	18.7(7.8-30.7)	ND	1 year: 67% 2 years: 33%. 3 years: 0%	—	TX	NP	NP	NP
**Melphalan**	Vogl[83]	2017	Retrospective case series (III)	18	7 (39%)	5 (28%)	NP	TX	9,6(range 1.6–41.0)	NP	1 year: 44%.	—	TX	12.4 (range 0.9–41.0)	NP	• Grade 3 or 4: during IHP: Hypotension (n = 2) (11.11%), Tachycardia (n = 1) (5.55%), Ventricular fibrillation (n = 1) (5.55%), Coagulopathy (n = 1)(5.55%)<br>• Grade 3 or 4: up to 30 days after IHP and temporary: Leukopenia (n = 11) (61.11%), Thrombocytopenia (n = 8) (44.44%), Fever (n = 4) (22.22%), Edema (n = 2) (11.11%), Infection (n = 2) (11.11%)

AE, adverse effect; CI, confidence interval;EP, enrollmentperiod ; ND, not defined; NP, not provided; LFT, liver function tests; OS, overall survival; TX, treatment.

Table S6. Included studies of Immunotherapy.

**Drug / Intervention**	**Author**	**Yearofpublication**	**Studio design**	**Numberofpatients**	**Number of first line treatments (%)**	**Number of patients with surgical [illegible]**	**Median from metastases to treatment in months**	**DefinitionofOS**	**Median OS published in months (range)**	**CIOS**	**AnnualOS**	**Exceptional outcomes**	**Definition Free Interval of Progression (FIP)**	**Median FIP**	**CI FIP**	**Adverse effects**
**Ipilimumab**<br>**Pembrolizumab**<br>**Ipilimumab /Nivolumab**	Bol [21]	2019	Retrospective case series (III)	24<br>43<br>19	24 (100%)<br>43 (100%)<br>19 (100%)	NP	NP	TX	9,9<br>10,3<br>18,9	NP	1 year: 50%<br>1 year: 38.7%<br>1 year: 57.6%	1 pacient alive until 46 months approx	TX	3<br>4,8<br>4	NP	NP
**Ipilimumab or Nivolumab or Pembrolizumab**	Xu[22]	2019	Retrospective case series (III)	18	18 (100%)	NP	NP	TX	15,8	NP	NP	—	TX	3	NP	NP
**Ipilimumab or Pembrolizumab or Nivolumab + HIA**	Itchins [50]	2017	Retrospective case series (III)	6	NP	NP	NP	DX	18,2	95% CI (10.9-NR)	NP	—	TX	7	CI 95% (2-NR)	• Pruritus 4 (66,7%), Hypothyroidism 2 (33,3%), Hypophysitis 2 (33%)
**Ipilimumab or Nivolumab or Pembrolizumab or Atezolizumab**	Klemen [85]	2020	Retrospective case series (III)	30	NP	NP	NP	TX	12,2	NP	1 year: 50%<br>5 years 22%	4 patients alive at 60 months	NP	NP	NP	NP
**Ipilimumab 3mg/ kg + RFA**<br>**Ipilimumab 3mg/ kg + RFA**<br>**Ipilimumab 10 mg/kg + RFA**	Rozeman [86]	2019	Phase Ib/II non-randomized prospective trial (IIa)	3<br>19<br>19	3 (100%)<br>19 (100%)<br>19 (100%)	0 (0%)<br>0 (0%)<br>0 (0%)	NP<br>NP<br>NP	EP<br>EP<br>EP	NP<br>9,7<br>14,2	NP<br>NP<br>NP	1 year: 66.7% 2 years: 33.3% 3 years: 33.3%<br>1 year: 42.1% 2 years: 0%<br>1 year: 57.9% 2 years: 10.5% 3 years: 5.3%	—	EP<br>EP<br>EP	NP<br>2,76<br>2,76	NP<br>NP<br>NP	• AE 18 (94.7%): Diarrhea 7 (37%), Fatigue 6 (32%), Nausea 6 (32%), Pruritus 6 (32%) Grade 3 or 4: 6 (32%): Colitis 5(26%)<br>• AE 18 (94.7%): Diarrhea 11 (58%), Rash 9 (47%), Fatigue 4 (21%), Pruritus 4 (21%) Grade 3or 4: 10 (52%): Colitis 9 (47%)

**Ipilimumab + NivolumabORPembrolizumab ***	Heppt[87]	2019	Multi-centre etrospective study (III)	64	50 (78.1%)	NP	NP	TX	16,1	CI 95% (12.9-19.3)	NP	—	TX	3	CI 95% (2.4–3.6)	• AE: 37 (60.9%)<br>• Grade 3 or 4: 25(39.1%): Colitis 13(20.3%), Hepatitis 13(20.3%), Thyroiditis 10(15.6%), Pituitary 5(7.8%)
**Ipilimumab + Nivolumab****	Karivedu [88]	2019	Retrospective case series (III)	8	7 (87,5%)	0 (0%)	NP	TX	14,2	CI 95% (1.9-24) ***	1 year: 62.5%*** 2 years: 12.5%***	—	NP	NP	NP	• Autoimmune Colitis 4 (50%)
**Pembrolizumab**	Rossi [89]	2019	Observational prospective cohort (III)	17	17 (100%)	0 (0%)	NP	TX	NR	NP	NP	—	TX	3,8	CI 95% (2.9-9.7)	• Grade 1: hypothyroidism 2(11.7%)<br>• Grade 2: hypophysistis 1(5.8%)<br>• No grade 3 and 4
**Nivolumab**	Namikawa [90]	2019	Retrospective case series (III)	14	6 (42,86%)	0 (0%)	NP	TX	13.8 (range 1.15-24.16)	NPᶲ	1 year: 66,6% 2 years: 14,2%	—	TX	2.3 (range, 1-24.2)	CI 95% (0-9,18)	• AE: Fatigue 8(57%), Pruritus 7(50%), Nausea 6(43%), Altered liver tests 6(43%)<br>• Grade 3 or 4: Hyperglycemia 1(7%), Dyspnea 1(7%), Pneumonitis 1(7%)
**Ipilimumab**	Yasar [91]	2019	Retrospective case series (III)	20	NP	NP	NP	NP	5	CI 95% (1-17)	NP	—	NP	NP	NP	NP
**Ipilimumab+Pembrolizumab**	Kirchberger [92]	2018	Retrospective case series (III)	9	1 (11%)	NP	NP	TX	18,4	NP	1 year: 77,7% 2 years: 22,2%	—	NP	NP	NP	NP
**Pembrolizumab**				54	35 (64,8%)				14					3,1		• AE: 14(25.9%)<br>• Grade 3 or 4: 4(7.4%): Arthritis 1(1.8%), Autoimmune Hepatitis 1(1.8%), Cardiac Toxicity 1(1.8%), Serum Lipase Elevation 1(1.8%)
**Nivolumab**	Heppt [93]	2017	Retrospective case series (III)	32	23 (71,9%)	NP	NP	TX	10	NP	NP	—	TX	2,8	NP	
**Ipilimumab+ (PembrolizumabORNivolumab)**				15	6 (40%)				NR					2,8		• AE: 13(40.6%)<br>• Grade 3 or 4: 4(71.4): Colitis 1(4.3%), Heart Toxicity 1(4.3%), Arthralgia 1(4.3%), Fatigue 1(4.3%)

																• AE: 7(46.7%)<br>• Grade 3 or 4: 2(13.3%): Thyroiditis, colitis, and pituitary disease
**NivolumaborPembrolizumab**	Bender [94]	2017	Retrospective case series (III)	15	6 (40%)	NP	NP	NP	5 (range 1-16)	NP	NP	—	NP	3 (range 0.75-6.75)	NP	• Autoimmune Hepatitis 1 (6.6%)
**Nivolumab + Ipilimumab + (Nivolumab as maintenance)**	Pelster[96]	2019	Prospective non-randomised, non-comparison phase II trial (IIb)	30	NP	NP	NP	TX	19,1	NP	1 year: 62%	—	NP	6	NP	• AE: 29(96.6%)<br>• Grade 3 or 4: 14(46.6%) (No detailed information)
**Pembrolizumabornivolumabor atezolizumab**	Algazi [97]	2016	Retrospective case series (III)	56	8 (14%)	0 (0%)	NP	TX	7,7	CI 95% (0.7-14.6)	NP	—	TX	2,6	CI 95% (2.4-2.8)	• Fatigue (19.6%), Pruritus (12.5%), Rash (10.7%), Nausea (10.7%), Hypothyroidism (7.1%), Diarrhea (8.9%)<br>• Grade 3 or 4: 7 (12.5%) Nausea, Vomiting, Hyperbilirubinemia, Fatigue, Colitis, Arthralgia and Lymphopenia.
**Ipilimumab**	Danielli [98]	2012	Prospective non-randomised Clinical Trial (IIa)	13	0 (0%)	NP	NP	TX	8,3 (range, 0.5-39.6)	CI 95% (4.2-12.4)	1 year: 30.8%<br>2 years:7.7%<br>3 years:7.7%	—	NP	NP	NP	• Grade 3 or 4: 3 (23%): Diarrhea 1 (7.70%), ALT Elevation 1 (7.70%), AST Elevation 1 (7.70%)
**Tremelimumab**	Joshua [99]	2015	Phase II Prospective Trial (IIb)	11	NPɸɸ	3 (27%)	NP	EP	12,8	CI 95% (3.8-19.7)	NP	—	TX	2,9	95% CI (2.8-3)	• Hyperthyroidism 2 (18.2%)<br>• Grade 3 or 4: Rash 1 (9.1%), Nausea 2 (18.2%) Diarrhea 3 (27.3%)
**Pembrolizumab**	Karydis [100]	2016	Retrospective case series (III)	25	0 (0%)	3 (12%)	11.3 (range 3.7-65.1)	TX	>7.4 (range, 0.2-16.4)	NR	1 year: > 28%	—	TX	3 (range, 0.3-10.6)	NR	• Grade 3 or 4: 5 (20%): Transaminitis 1 (4%), Rash and itching 1 (4%), Pituitary 2 (8%), Fatigue 1 (4%), Diarrhea 1 (4%)

**Ipilimumab**	Kelderman [101]	2013	Retrospective case series (III)	22	0 (0%)	NP	NP	TX	5,2	CI 95% (4.9-9.6)	1 year: 27%	—	TX	2,9	CI 95% (2.3-5.3)	• Grade 3 or 4: 5 (22.72%), Colitis 2 (9.1%), Hepatitis 1 (4.5%) Other 3 (13.6%)
**Ipilimumab**	Luke [102]	2013	Retrospective case series (III)	39	0 (0%)	NP	NP	TX	9,6	CI 95% (6.3-13.4)	NP	—	NP	NP	NP	• Rash 11 (28.21%), Thyroiditis 4 (10.26%)<br>• Grade 3 or 4: Diarrhea and colitis 2 (5.13%), Hepatitis and pancreatitis 1 (2.56%), Hypophystis 2 (5.13%), Uveitis 1 (2.56%)
**Ipilimumab**	Maio [103]	2013	Prospective non-randomised clinical trial (IIa)	83ϕϕϕ	0 (0%)	NP	NP	NP	6	CI 95% (4.3-7.7)	1 year: 31%	—	NP	3,6	CI 95% (2.8-4.4)	• Any grade: Rash 8 (10%), Pruritus 11 (13%)<br>• Grade 3 or 4: Toxic Hepatitis 2(2.5%), Diarrhea 2 (2.5%)
**NivolumaborPembrolizumab**	Van derKooij [104]	2017	Retrospective case series (III)	17	8 (47%)	NP	NP	TX	8,8	CI 90% (5.5-12.2)	NP	—	TX	2,3	CI 95% (1.7-3)	• Toxicodermia grade 2: 1 (5.88%)
**Ipilimumab**	Zimmer [105]	2015	Phase II Prospective Trial (IIb)	53	8 (15%)	NP	NP	TX	6,8	CI 95% (3.7-8.1)	1 year: 22% (CI 95% 12–35)<br>2 years: 7% (CI 95%1–18)	—	TX	2,8	CI 95% (2.5-2.9)	• Any grade: 35 (66%): Diarrhea 16 (30%), LFT 7 Alteration (13%), Colitis 9 (17%), Pruritus 5(9%), Rash 3 (6%)<br>• Grade 3 or 4: 19 (36%): Diarrhea 7 (13%), LFT alteration 4 (8%), Colitis 6 (11%)
**Nivolumab+Ipilimumab**	Piulats Rodriguez [106]	2018	Prospective non-randomised, non-comparison phase II trial (IIb)	50	50 (100%)	NP	NP	TX	12,7	NP	NP	—	TX	3,27	NP	• AE: 46(92%)<br>• Grade 3 or 4: 27(54%), Increase in liver enzymes, Dermatological pathology, Hepatitis, Diarrhea.

Approx, approximate; DX, diagnosis; AE, adverse effect; CI, confidence interval; CPI, checkpoint inhibitors; NC, no prior chemotherapy or systemic treatment; NP, not provided; NR, not reached; LFP, liver function tests; EP, enrollment period; R-P, response-progression; OS, overall survival; TX, treatment. *Ipilimumab 3 mg/kg + nivolumab 1 mg/kg every 3 weeks, followed by nivolumab 3 mg/kg every 2 weeks. Ipilimumab 1 mg/kg + pembrolizumab 2 mg/kg every 3 weeks, followed by pembrolizumab 2 mg/kg every 3 weeks. ** Combined with TACE. *** Calculated from the narrative of the manuscript. ϕ CI 95% of the median overall survival was

not reached; i.e. cumulative survival plot did not fall below 50%. ϕϕThree patients had undergone surgery, three palliative radiation therapy, one TACE, three systemic chemotherapy. ϕϕϕ One of the 83 patients was lost to follow-up, so the Kaplan-Meyer graph represents 82.

Table S7. Included Targeted Therapy Studies.

**Drug / Intervention**	**Author**	**Yearofpublication**	**Studio design**	**Numberofpatients**	**Number of first line treatments (%)**	**Number of patients with surgical [illegible]**	**Median from metastases to treatment in months**	**DefinitionofOS**	**Median OS published in months (range)**	**CIOS**	**AnnualOS**	**Exceptional Outcomes**	**Definition Free Interval of Progression**	**Median FIP**	**CI FIP**	**Adverse effects**
**MAPK INHIBITORS**																
**Cabozantinib**	Luke [23]	2019	Phase II Prospective Randomized Clinical Trial (Ib)	30	11 (63.3%)	NP	NP	TX	6.3	CI 95% (5.5-10.3)	NP	—	TX	2	CI 95% (1.8-5.3)	• AS 30 (100%): Increase AST 18 (58.1%) Increase ALT 18 (58.1%) Fatigue 14 (45.2%) HTA 14 (45.2%)<br>• Grade 3 or 4: 16 (51.6%): Fatigue 3(9.7%) ETS 3 (9.7%) Bilirubin 2 increase (6.5%) Thromboembolic event 4(12.9%)
**Selumetinib + Dacarbazine**	Carvajal [39]	2018	Phase III Prospective Randomized Clinical Trial (Ib)	97	97 (100%)	0 (0%)	NP	EP	10 (approx.)	NP	NP	—	EP	2.8 (approx.)	NP	• AE 97 (100%): Nausea 60(62%), Rash 55(57%), Fatigue 43(44%), Diarrhea 43 (44%).<br>• Grade 3 or 4: 60 (61%): Neutropenia 19 (19%), Thrombocytopenia 12(12%), ETS 8(8%), LFT alteration 6(6).
**Sunitinib**	Mahipal [112]	2012	Prospective non-randomised pilot study (IIa)	20	3 (15%)	3 (15%)	NP	TX	8,2	NP	1 year: 44.4% CI 95% (22.5-64.4)	—	TX	4,2	NP	• Any AE: Fatigue 18(90%), Diarrhea 12(60%), Bleeding 11(55%), Anorexia 9(45%)<br>• Grade 3: Fatigue 3(15%), Leucopenia 2(10%),

																Anorexia 2(10%), Vomiting 2(10%)
**Sorafenib + Carboplatin + Paclitaxel**	Bhatia [113]	2012	Phase II Prospective Trial(IIb)	24	20 (83%)	NP	NP	EP	11	95% CI (7-14)	1 year: 42% CI 95% (22-60)	___	EP	4	95% CI (1-6)	• Grade 3: 12(50%); Neutropenia 4(16.6%), Thrombocytopenia 3(12.5%), Diarrhea 2(8.3%), Pruritus 1(4.1%)<br>• Grade 4: 7(29%); Neutropenia 6(25%), Lymphopenia 2(8.3%), Febrile Neutropenia 1(4.1%)
**Sorafenib**	Mouriaux [114]	2016	Phase II Prospective Trial(IIb)	32	19 (59%)	0 (0%)	4,9 (range 0-8)	TX	NP	NP	NP	___	NP	NP	NP	• Any AE: Fatigue 19(59.4%), Diarrhea 18(56.2%), Nausea and Vomiting 17(53.1%), Hand and Foot Syndrome 17(53.1%)<br>• Grade 3 or 4: 10(34.3%); HTA 4(12.5%), Hand Foot Syndrome 2(6.2%), Rash 1(3.1%), Alopecia 1(3.1%)
**Sorafenib+Fotemustine**	Niederkorn [115]	2014	Retrospective case series (III)	8	2 (25%)	NP	NP	TX	15,9 (range, 5,6-29,6)	CI 95% (11,7-20,1)	1 year: 75%<br>2 years: 12,5%	___	NP	NP	NP	• Hematological AE grades 1-3: 8(100%)<br>• Neutropenia and / orthrombicytopenia: 3 (37,5%)<br>• Hand-foot syndrome and / or maculopapular rash 4 (50%)
**Imatinib**	Hofmann [116]	2009	Prospective non-randomised Clinical Trial (IIa)	12	8 (67%)	NP	NP	TX	6,8**	NP	NP	___	NP	NP	NP	• Grade 3: Fatigue 4 (33%), Myalgia 2 (16,6%), Abdominal pain 3 (25%), Vomiting 7 (58,3%), Facial edema 2 (16,6%)<br>• Grade 4: Vomiting 1 (8,3%), Anemia 1 (8,3%)

**Imatinib mesilate**	Penel [117]	2008	Phase II Prospective Trial(IIb)	13	6 (46%)	0 (0%)	NP	TX	10,8	NP	NP	—	NP	NP	NP	• Any AE: 13(100%)<br>• Grade 3 and 4: 5(38.5%); Hepatic cytolysis 3(23.1%), Vomiting 2(15.4%), Abdominal pain 2(15.4%), Myalgia 1(7.6%)
**Cabozantinib**	Daud [118]	2017	ProspectiveRandomized Trial (Ib)	23	NP*	NP	NP	TX	12,6	NP	NP	—	TX	4,8	NP	NP*
**HSP90 INHIBITOR**																
**STA-9090 (Ganetespib)**				17	NP	NP	NP		7	CI 90% (4.0-13.0)				1,8	CI 90% (1,2-3,5)	• AE: 17 (100%) Increase in transaminases, nausea, vomiting, diarrhea
**STA-9090 (Ganetespib) 200 mg / 1 time per week**	Shah [111]	2018	Phase II non-randomised controlled open-label Clinical trial (IIa)	7	NP	NP	NP	TX	8.5	CI 90%. (3.7 - 28.7)	NP	—	TX	1,6	CI 90% (0.8-3.5)	• Grade 3: 11(64.7%)
**STA-9090 (Ganetespib) 150mg / 2 times a week**				10	NP	NP	NP		4.9	CI 90%. (3.2 - 13.0)				1,8	CI 90% (0.9-7.1)	• Grade 4: 2 (11.7%)

Approx, approximate; AE, adverse effect; CI, confidence interval; EP, enrollment period; NP, not provided; OS, overall survival; TX, Treatment. * Provided the percentages of all patients, not uveal melanomas specifically. ** The median survival seems to correspond to the arithmetic mean.

Table S8. Included Selective Internal Radiation Therapy (SIRT).

**Drug / Intervention**	**Author**	**Yearofpublication**	**Studio design**	**Numberofpatients**	**Number of first line treatments (%)**	**Number of patients with surgical treatment (%)**	**Median from metastases to treatment in months**	**DefinitionofOS**	**Median OS published in months (range)**	**CIOS**	**AnnualOS**	**Exceptional Outcomes**	**Definition Free Interval of Progression**	**Median FIP**	**CI FIP**	**Adverse effects**

Radioembolization, (Y-90) beads	Tulokas [24]	2018	Case Study Series comparative (III)	18<br>14<br>4	14 (77,7%)<br>14 (100%)<br>0 (0%)	0 (0%)<br>0 (0%)<br>0 (0%)	NP	TX	13.5 (range, 3.6-44.8)<br>18.7 (range, 8.2-44.8)<br>7.8 (range, 3.6-20.7)	95% CI (4.4-21)<br>95% CI (0-33)<br>ND	1 year: 55.5%<br>2 years: 27.7%<br>3 years:11.1%<br>1 year: 64.3%<br>2 years: 35.7%<br>3 years:14.3%<br>1 year: 25%<br>2 years: 0%<br>3 years: 0%	—	TX	5.6 (range, 1.3-40.8)<br>5.6 (range, 1.3-40.8)<br>3.7 (range, 4.4-5.6)	95% CI (3.6-5.6)<br>95% CI (0.4-11.4)<br>95% CI (2.8-4.6)	• Elevation of transaminases: grades 1-2: 11(61.1%) grades 3 and 4: 2(11.1%)<br>• Clinical grade 1-2: 15(83.33%) Nausea, abdominal pain, fatigue, fever.
Radioembolization, resin beads (Y-90) + Immunotherapy*<br>Radioembolization, resinbeads (Y-90)	Levey [125]	2019	Retrospective case series (III)	12<br>12	NP	NP	NP	TX<br>TX	26<br>9,5	CI 95% (18.9-67.9)<br>CI 95% (5.5-19.7)	1 year: >50% approx 2 years: >40 approx**<br>1 year: 40% approx 2 years: 20 approx**	1 patient alive until 95 months** 1 patient alive until 120 months**<br>—	TX<br>TX	10.3<br>2.7	CI 95% (6.1-26.3)<br>CI 95% (1.8-5.2)	NP
Radioembolization, resin or glass beads (Y-90)	Ponti [126]	2019	Retrospective case series (III)	22	22 (100%)	0 (0%)	3 (range 1-5)	TX	18	95% CI (8-28)	NP	—	TX	5	CI 95% (1.84-17)	• Grade 1 and 2: 9(41%) Abdominal pain and fatigue<br>• Grade 3: 7(32%) Cholecystitis 3(13.6%), Abdominal pain 2(9.1%) ,Liver failure 2(9.1%)<br>• Grade 4: Non-referral
Radioembolization, resin beads (Y-90)<br>Radioembolization, resin beads (Y-90) post Immuno-embolization and progression	Gonsalves [127]	2019	Phase II Prospective Non-Randomised Trial (IIa)	24<br>24	24 (100%)<br>0 (0%)	NP	NP	TX	18,5 (range 6.5-73.7)<br>19,2 (range 4.8–76.6)	CI 95% (11.3-23.5)<br>CI 95% (11.5-24)	1 year: 60.9%<br>2 yeras: 20.1%<br>3 years: 13%<br>1 year: 69.6%.<br>2 yeras: 30.4%<br>3 years: 8.7%	—<br>—	TX	8,1 (range 3.3 – 33.7)<br>5,17 (range 2.9–22.0)	CI 95% (6.4-11.8)<br>CI 95% (3.7-9.8)	• Fatigue 22 (91.7%) Increase in liver enzymes 19 (79.2%) Abdominal pain 14 (58.3%) Nausea and vomiting 14 (58.3%)<br>• Grade 3: 3(12.5%): Abdominal pain 2 (8.3%) and Nausea and vomiting 1 (4.1%), Fatigue 17(70.8%) Liver enzyme increase 21(87.5%) Nausea and vomiting 14(58.3%)

Radioembolization, resin beads (Y-90) + Immunotherapy (Nivolumab or Pembrolizumab or Ipilimumab)	Zheng [128]	2018	Retrospective case series (III)	11	2 (18,18%)	NP	9 (range, 2-37,5)	TX<br>DX	17<br>35,5	CI 95% (1.8-32.2)<br>CI 95% (10-55)	1 year: 50%	—	TX	15	CI 95% (5.9-24.1)	• Grade 1 or 2: 10 (90.9%): Fatigue, nausea, anorexia, abdominal pain, altered liver tests<br>• Grade 3: 1 (9%) pepticulcer
Radioembolization, resinbeads (Y-90)	Xing [129]	2017	Case Study Series comparative (III)	15	0 (0%)	NP	NP	TX	10,9	CI 95% (8.5-13.4)	NP	—	NP	NP	NP	NP****
Radioembolization, resinbeads (Y-90)	Eldredge-Hindy [130]	2016	Retrospective case series (III)	50	13 (18%)***	NP	9,8	TX	12,3 (range, 1,9-49,3)	NP	NP	—	TX	5,9 (range, 1,3-19,1)	NP	• Grades 1 and 2: Fatigue 31(44%), Abdominal pain 18(25%), Nausea 11(15%)
Radioembolization, resinbeads (Y-90)	Klingenstein[131]	2013	Retrospective case series (III)	13	2 (15%)	1 (8%)	5 (range 1-49)	TX	7 (range, 2-25)	ND	1 year: 46,1%<br>2 years: 7,6%	—	NP	NP	NP	• Grades 1 and 2: Abdominal pain 5(38%), Nausea 3(23%), Gastric ulcer 1(7.6%)<br>• Grade 4: Hepatomegaly and death from liver failure 1 (7.6%)
Radioembolization, resinbeads (Y-90)	Schelhorn[132]	2015	Retrospective case series (III)	8	0 (0%)	0 (0%)	17.1 (range 6.4-23.2)	TX	2,8(range, 0,85-14,4)	NP	NP	—	TX	0.98 (range 0.78-6.6)	NP	• Grade 2: Nausea 1 (12.5%)<br>• Grades 3 and 4: 0 (0%)

CI, confidenceinterval; NP, not provided; ND, not defined; EP, enrollment period; OS, overall survival; TX, treatment. * Immunotherapy 3 months before or after the Radioembolization Drugs: Ipilimumab, Nivolumab, Pembrolizumab or Il-2. **Kaplan-Meier diagram estimated data. *** Pre-treatment was reported for all 58 patients, not specifically for the 50 patients who had a pre-treatment PET/CT scan and were included in the Kaplan-Meier diagram. **** Provided the percentages of all patients, not uveal melanomas specifically.

Table S9. Included Immunoembolisation Studies (IE).

**Drug / Intervention**	**Author**	**Yearofpublication**	**Studio design**	**Numberofpatients**	**Number of first line treatments (%)**	**Number of patients with surgical**	**Median from metastases to treatment in months (range)**	**DefinitionofOS**	**Median OS published in months (range)**	**CIOS**	**AnnualOS**	**Exceptional Outcomes**	**Definition Free Interval of Progression (FIP)**	**Median FIP**	**CI FIP**	**Adverse effects**

**Immunoembolisationwith GM-CSF**	Valsecchi [73]	2015	Phase II Prospective Randomized Trial (Ib)	25	NP	NP	NP	TX	21,5	CI 95% (18.5-24.8)	NP	1 patient still alive at 50 months	TX	10,4	CI 95% (7,5-13,2)	• First week: Abdominal pain (51.6%), LFT alteration (19.9%), Haematological (2.1%)<br>• Grade 3 or 4: LFT alteration (2.7%), Hematological (0.8%)<br>• From the second week onwards: Abdominal pain (20.1%), LFT alteration (12.2%), Haematological (11.2%)<br>• Grade 3 or 4: Lessthan 1%
**Immunoembolisationwith GM-CSF**	Sato [133]	2008	Phase I Prospective Trial (IIb)	34	28 (82%)	3 (9%)	NP	TX	14.4*	CI 95% (11.2-22.3)	1 year: 61% CI 95% (45-78.1)<br>2 years: 26% CI 95% (11.2-41)	1 patient was alive at 40.8 months of follow-up	TX	Hepatic: 4.8<br>Extrahepatic: 10.4	CI 95% (3.6-11.5)<br>CI 95% (6.8-12.4)	• Grade 3: Liver enzyme elevation 3(8.8%), abdominal pain 1(2.9%).<br>• Grade 4: Respiratoryfailure 1(2.9%)

CI, confidence interval; NP, not provided; LFT, liver function tests; OS, overall survival; TX, Treatment. * Median overall survival is reported for 34 patients by intention-to-treat analysis, while the Kaplan-Meier diagram includes 31 radiographically evaluable patients.

Table S10. Included studies of Immunosuppressant (IS).

**Drug / Intervention**	**Author**	**Yearofpublication**	**Studio design**	**Numberofpatients**	**Number of first line treatments (%)**	**Number of patients with surgical treatment**	**Median from metastases to treatment in months (range)**	**DefinitionofOS**	**Median OS published in months (range)**	**CIOS**	**AnnualOS**	**Exceptional Outcomes**	**Definition Free Interval of Progression (FIP)**	**Median FIP**	**CI FIP**	**Adverse effects**
**Everolimus+Pasireotide**	Shoushtari [139]	2016	Phase II Prospective Trial(IIb)	14	0 (0%)	NP	NP	TX	11 (range, 4.5-28.5)	NP	NP	—	TX	3.7 (range 1.6-7.6)	NP	• Any AE: 14 (100%); Hyperglycemia 12(85.5%), Hypertriglycemia 10(71.4), Diarrhea 9(64.2%), Leukopenia 9(64.2%)<br>• Grade 3: Hyperglycemia 7(50%), Oral Mucositis

2(14.2%), Diarrhea 1(7.1%), Hypokalemia 1(7.1%)

AE, adverse effects;CI, confidence interval; NP, not provided; OS, overall survival; TX, Treatment.

Table S11. Included studies of Liver-directed thermotherapy (LDT).

Drug / Intervention	Author	Yearofpublication	Studio design	Numberofpatients	Number of first line treatments (%)	Number of patients with surgical treatment (%)	Median from metastases to treatment in months	DefinitionofOS	Median OS published in months (range)	CIOS	AnnualOS	Exceptional Outcomes	Definition Free Interval of Progression (FIP)	Median FIP	CI FIP	Adverse effects
**Radiofrequencystereotacticablation**	Bale [140]	2016	Retrospective case series (III)	6	0 (0%)	0 (0%)	NP	TX	38	NP	NP	—	NP	NP	NP	For all patients (6 uveal, 14 cutaneous)<br>• No procedure-related deaths<br>• In 34 SRFA sessions,a total of 3 complications (3 pleural effusions)
**Laser-inducedThermotherapy (LITT)**	Eichler[141]	2014	Retrospective case series (III)	18	NP**	4 (22%)	NP	TX	33.6	CI 95% (12-60)	1 year: 88%<br>3 years: 47%.<br>5 years: 18%	—	NP	NP	NP	• Pain instead of injection18(100%), Asymptomatic pleural effusion 2(11.1%)

CI, confidence interval;NP, not provided; OS, overall survival; TX, treatment. *The authors state: "The limitations of our study are the small number of patients and the non-homogeneous population with respect to various pre-LITT treatments such as immunochemotherapy and TACE".

Table S12. Included Dendritic Cell Vaccine Studies.

Drug / Intervention	Author	Yearofpublication	Studio design	Numberofpatients	Number of first line treatments (%)	Number of patients with surgical treatment (%)	Median from metastases to treatment in months (range)	DefinitionofOS	Median OS published in months (range)	CIOS	AnnualOS	Exceptional Outcomes	Definition Free Interval of Progression (FIP)	Median FIP	CI FIP	Adverse effects
**Dendriticcellvaccine**	Bol[143]	2014	Prospective non-randomised comparative case series (IIa)	14	8 (57%)	3 (21%)	NP	TX	19,2	95% CI (3.1-34.9)	1 year: 64% 2 years: 50% 3 years: 29%	1 patient alive at 84 months	NP	NP	NP	• Grade 1 and 2: Flu-like symptoms 8(57.1%), Erythema 6(42.8%), Fatigue 5(35.7%). • Grade 3 and 4: 0(0%)

CI, confidence interval; NP, not provided; OS, overall survival; TX, Treatment.

Table S13. Included studies of CIOVIR-5 Oncolytic Adenovirus.

Drug / Intervention	Author	Yearofpublication	Studio design	Numberofpatients	Number of first line treatments (%)	Number of patients with surgical treatment (%)	Median from metastases to treatment in months	DefinitionofOS	Median OS published in months (range)	CIOS	AnnualOS	Exceptional Outcomes	Definition Free Interval of Progression	Median FIP	CI FIP	Adverse effects
**CIOVIR-5, intravenousoncolytic adenovirus**	Garcia [146]	2018	Phase I Clinical trial IIb	6	NP	NP	NP	NP	8,9	NP	NP	___	NP	NP	NP	• Grade 3 Toxicity: 2(33.3%) Neutropenia 1 (16.6), Increased Transaminase 1 (16.6), Thrombocytopenia 1 (16.6)

CI, confidence interval; NP, not provided; OS, overall survival.

Table S14. Included studies of Surgical Resection.

Drug / Intervention	Author	Yearofpublication	Studio design	Numberofpatients	Number of first line treatments (%)	Number of patients with surgical	Median from metastases to treatment in months	DefinitionofOS	Median OS published in months (range)	CI OS	AnnualOS	Excepcional Outcomes	Definition Free Interval of Progression (FIP)	Median FIP	CI FIP	Adverse effects

Resection+ fotemustine or dacarbazine IA+ cisplatin IA<br>CurativeResection<br>Tumourreduction	Salmon [57]	1998	Prospective non-randomized comparative case series (III)	19<br>34	19 (100%)<br>34 (100%)	0 (0%)<br>0 (0%)	NP	NP	22<br>NP	NP	NP	—	NP	NP	NP	NP
Resection	Hsueh [148]	2004	Comparative retrospective case series (III)	24	NP*	NP	NP	DX	38**	NP	5 years: 39%	8 patients still alive at 60 months	NP	NP	NP	NP
Resection<br>R0<br>R1/R2	Frenkel [149]	2009	Comparative retrospective case series (III)	14<br>23	NP	0 (0%)<br>0 (0%)	NP	TX<br>TX	65.6 (range 11.5 to NP)<br>16.6 (range. 7.6-25.5)	NP	NP	—	NP	NP	NP	NP
Resection	Aoyama [150]	2000	Retrospective case series (III)	12	8 (57%)	0 (0%)	NP	TX	27	CI 95% (25.7-28.3)	5 years: 53.3%.	2 patients still alive at 60 months	TX	19 (range 6-78)	NP	NP
Resection + fotemustine IA or cisplatin IA<br>R0<br>R2	Kodjikian [151]/ Rivoire [152]	2005	Comparative retrospective case series (III)	14<br>14	14 (100%)<br>14 (100%)	0 (0%)<br>0 (0%)	NP	TX<br>TX	25**<br>16	NP	2 years: 59.9%<br>2 years: 14.3%.	----	NP	NP	NP	NP
Resection + FEP	Servois [153]	2019	Phase II Prospective Non-Randomised Trial (IIa)	14	14 (100%)	0 (0%)	NP	DX	NP	NP	5 years: 70% 10 years: 35%	10 patients still alive at 60 months	NP	NP	NP	NP
Resection***	Gomez [154]	2014	Comparative retrospective case series (III)	18	NP	NP	NP	TX	27(range, 14-90)**	NP	1 year: 100% 3 years: 41.4%. 5 years: 41.4%.	2 patients still alive at 60 months	NP	NP	NP	• Any AE: 4(23.5%)<br>• Grade 2: 3(16.6%)<br>• Grade 3: 1(5.5%)<br>• Grade 4: 0(0%)

Resection	Mariani [155]	2016	Comparative retrospective case series (III)	57	57 (100%)	0 (0%)	NP	TX	27	NP	NP	___	TX	10	NP	NP
Resection + FEP				13	13 (100%)	0 (0%)		TX	28				TX	7		
PartialResection[ϕ]	Yang [156]	2013	Retrospective case series (III)	5	NP	NP	NP	TX	11,5	CI 95% (7.5-15.8)	1 year: 40%<br>2 years: 25%<br>3 years: 0%	___	TX	5.5 (range, 4.5-14)	NP	• Pleural effusion 1 (20%)
Resection[±]	Mariani [157]	2009	Comparative retrospective case series (III)				68 (range, 19-81)			NP		Global survival at 60 months: 7%	NP	NP	NP	NP
R0				76	76 (100%)	0 (0%)		TX	27		2 years: 54.4%<br>3 years: 30%					
R1				22	22 (100%)	0 (0%)		TX	17		2 years: 30.7%<br>3 years: 13.6%					
R2				157	157 (100%)	0 (0%)		TX	11		2 years: 14.4%.<br>3 years: 5.7%					

AE, adverse effect; CI, confidence interval; DX, diagnosis; EP, enrollment period; IA, intra-arterial; NP, not provided; OS, overall survival; RFA, radiofrequency ablation; TX, treatment. * The authors state: "Postoperative chemotherapy was mandatory in all patients during the first years of our experience, but since 2005 it has been reserved for patients randomised in the intra-arterial arm of the European trial"; in addition, eight patients underwent RFA during laparotomy to achieve R0 resection. ** The reported median survival seems to be calculated arithmetically and not by a Kaplan-Meier estimate. *** A patient received RFA. ±The authors state: "Some patients received chemotherapy intravenously or through an intra-arterial liver port, and some received adjuvant immunotherapy. ϕ Of the five patients, two received adjuvant TACE therapy with or without immunotherapy.
